# Design of a large-range rotary microgripper with freeform geometries using a genetic algorithm

**DOI:** 10.1038/s41378-021-00336-0

**Published:** 2022-01-06

**Authors:** Chen Wang, Yuan Wang, Weidong Fang, Xiaoxiao Song, Aojie Quan, Michiel Gidts, Hemin Zhang, Huafeng Liu, Jian Bai, Sina Sadeghpour, Michael Kraft

**Affiliations:** 1 College of Optical Science and Engineering, Zhejiang University, Hangzhou, China; 2 Department of Electrical Engineering and Computer Science, University of Liege, Liege, Belgium; 3 ESAT-MNS, University of Leuven, Leuven, Belgium; 4 PGMF and School of Physics, Huazhong University of Science and Technology, Wuhan, China

**Keywords:** Electrical and electronic engineering, Micro-optics

## Abstract

This paper describes a novel electrostatically actuated microgripper with freeform geometries designed by a genetic algorithm. This new semiautomated design methodology is capable of designing near-optimal MEMS devices that are robust to fabrication tolerances. The use of freeform geometries designed by a genetic algorithm significantly improves the performance of the microgripper. An experiment shows that the designed microgripper has a large displacement (91.5 μm) with a low actuation voltage (47.5 V), which agrees well with the theory. The microgripper has a large actuation displacement and can handle micro-objects with a size from 10 to 100 μm. A grasping experiment on human hair with a diameter of 77 μm was performed to prove the functionality of the gripper. The result confirmed the superior performance of the new design methodology enabling freeform geometries. This design method can also be extended to the design of many other MEMS devices.

## Introduction

Microelectromechanical system (MEMS) microgrippers are microscale grippers fabricated through a micromachined process, and typically comprise actuators, mechanical parts for the handling and manipulation of micro-objects (1–100 μm) and force sensors. MEMS microgrippers are widely used in handling cells and tissues in biology^1^ and in microassembling and testing the mechanical properties of micromachined devices^2^.

MEMS microgrippers with different shapes, actuation, and sensing principles have been developed in recent years. The designs reported in refs. ^3,4^ were thermally actuated microgrippers. These microgrippers have a large displacement and a low actuation voltage. However, the high working temperature of thermally actuated microgrippers can be harmful to living cells and tissues in biological manipulation. Another design described in ref. ^5^ was based on a piezoelectric actuated microgripper. Although this design features a large displacement and bandwidth, it requires a complicated fabrication process and exhibits hysteresis nonlinearity, which severely limits its spatial resolution during manipulation.

Moreover, piezoelectric actuated microgrippers cannot work in a high-temperature environment. A magnetically actuated gripper was reported in ref. ^6^. This design provides a large displacement and a quick response with reasonable sensitivity, but it requires a complicated and expensive assembly process. Alternatively, a microgripper based on a shape memory alloy was discussed in ref. ^7^. This design had excellent flexibility and large bandwidth. However, it also suffered hysteresis nonlinearity and large power consumption. Electrostatically actuated microgrippers were reported in refs. ^8,9^. In particular, for the first time, Chang et al. introduced a rotary actuation comb into an electrostatically actuated microgripper to increase the displacement range to 94 μm with an actuation voltage of 100 V^10^. These designs feature a fast response time, low power consumption and no hysteresis. However, these designs have a relatively large dimension due to the high number of actuation comb fingers required. In addition, the maximum displacement of the electrostatically actuated microgripper is limited by the pull-in effect^11^. In addition, the actuation voltage of the electrostatically actuated microgrippers is relatively high, and normally, a voltage larger than 80 V is required to achieve a displacement of 100 μm. Such a high actuation voltage is not only problematic in practical applications but can also damage gripped samples.

In the vast majority of MEMS devices, simple geometrical layouts comprising only a few basic building blocks, such as beams, rectangular masses, and, more rarely, rings or disk-shaped structures, are used^12^. As discussed in the following, there are cases in which such conventional, simple designs limit the performance of MEMS devices and therefore may not meet the requirements for specific applications. Compared with conventional designs, geometries comprising more complex geometries offer a designer more freedom. Complex geometries may result in novel designs with superior performance^13^ and overcome the limitation of simple mechanisms^14–17^. For example, by using curved anti-springs, Middlemiss et al.^14^ and Boom et al.^15^ developed MEMS accelerometers with resolutions at the nano-g level. These anti-springs feature a low effective spring constant that cannot be achieved with conventional orthogonal designs under the same fabrication constraints. However, complex theoretical calculations are needed to design these complex geometries. Such a design method requires considerable design expertise and is practically impossible to transfer to other devices; a case-by-case approach is required. An alternative is topology optimization, which can be used to design MEMS devices with complex geometries. Ananthasuresh et al.^16,17^ and Seshia et al.^18^ developed complex force and motion amplification mechanisms to increase the sensitivity of accelerometers. Cao and Zhang et al. developed a module optimization method as a unified design approach for both compliant mechanisms and rigid-body mechanisms^19^. In the module optimization approach, the states of joints and links are fully parameterized, with which a designer can obtain a rigid-body mechanism, a partially compliant mechanism, or a fully compliant mechanism for a given design objective. However, in these MEMS devices, simple beam (or truss) elements are typically used as a fundamental building block to form optimized topologies. Such methodology easily results in designs that often cannot be fabricated since it is difficult to implement fabrication constraints well in the topology optimization process^18^.

In this paper, we introduce a novel electrostatically actuated microgripper with freeform geometries designed by a genetic algorithm (GA) approach. The novel design approach is introduced by describing the optimization process for a microgripper as a case study. In our previous work, a GA was introduced for the first time for the design of freeform geometries for MEMS sensors. Specifically, a MEMS accelerometer comprising a mechanical motion amplifier was described to demonstrate the effectiveness of the design approach^20^. In the following, we describe a MEMS actuator (i.e., microgripper) with freeform geometries that are designed and optimized by the GA-based design method. Due to the freeform geometries, the designed microgripper features a large displacement with a low actuation voltage compared with previously described electrostatic microgrippers. Detailed theoretical analysis and experimental validation are conducted. A manipulation experiment using the designed microgripper for grasping human hair is shown. Moreover, the pull-in effect in electrostatically actuated microgrippers is also discussed. The performances of the designed microgrippers are compared with those of existing microgrippers.

## Design of the microgripper with freeform geometries

### A design methodology based on a genetic algorithm

**Fig. 1:**
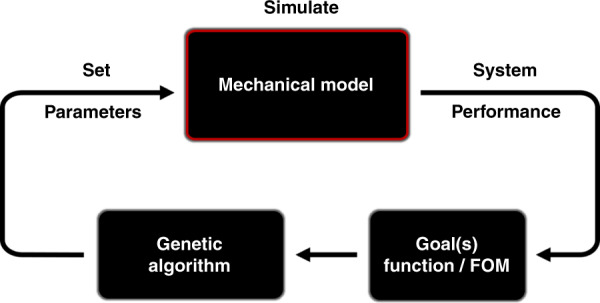
Optimization system. Generic process flow of the novel designed method with freeform geometries based on a GA

The microgripper in this work was designed using a novel design method allowing freeform geometries based on a GA. The methodology comprises two parts: a parametrized mechanical finite element model (FEM) with freeform geometries implemented in COMSOL^21^ and a GA implemented in MATLAB^21^, illustrated by the flow chart in Fig. 1. The FEM model and simulation in COMSOL can be directly controlled by MATLAB through LiveLink for MATLAB^21^. A GA is based on the mechanics of natural selection and genetics, combining the fittest individuals in the population to search for the best solution. These evolutionary-based techniques are excellent for particularly complex, multiparameter problems for which they are capable of finding good solutions in a short period of time. For optimization, the GA sets the parameter values of a mechanical model and simulates each “individual” parameter set in the first generation. Using a performance goal (or figure of merit (FOM)) function, the GA generates a new parameter set for the next generation. After several generations, the parameter values converge, indicating that the mechanical model reaches an optimal design. The details of the design process are described in the following.

### Microgripper model with freeform geometries

**Fig. 2:**
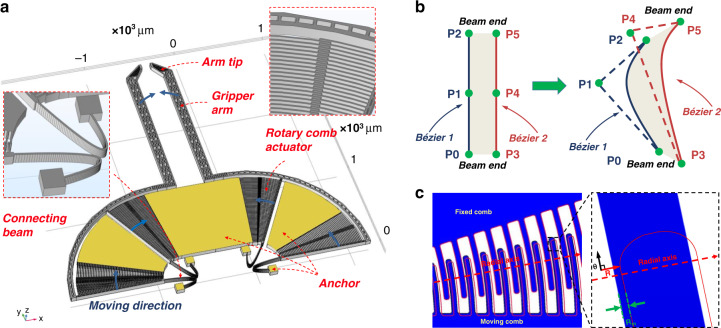
Proposed microgripper with freeform geometries and misalignment of rotary microgrippers. **a** Schematic view of the proposed microgripper with freeform geometries showing an arm tip, gripper arm, rotary comb actuator, connecting beam (freeform geometries), and anchor. The moving direction of the microgripper is along the direction of blue arrows. **b** With the use of Bezier curves, any beam can be defined with coordinates of six points. An orthogonal beam can be modified into a curved beam easily by just modifying the coordinates of P1 and P4. **c** Movement of a rotary comb actuator after actuation. The red line is the position of the moving comb before actuation, while the solid blue part is the position of the moving comb after actuation. The *R*-axis is along the radial axis of rotary comb fingers, which is the undesired displacement of comb fingers. The *θ*-axis is along the tangent direction of rotary comb fingers (perpendicular to the *R*-axis), which is the desired movement direction of comb fingers. The undesired movement along the radial axis, i.e., *R*_*m*_, reduces the gap between the fixed and moving comb fingers

A schematic drawing of a microgripper with freeform geometries is shown in Fig. 2a. It comprises rotary comb actuators, two gripper arms, two-arm tips to grasp micro-objects, and connecting beams that link the moveable structures to anchors. The gap between the two arm tips is 100 μm. When a voltage is applied to the rotary comb actuators, due to electrostatic force, the microgripper will move in the direction of the blue arrows, as indicated in Fig. 2a. This displacement is mechanically amplified and transferred to the arm tips through the gripper arms; this effectively functions as a mechanical lever^10^. The critical part of the microgripper is the connecting beam. It defines the total stiffness of the structure, which influences the actuation voltage, actuation displacement, bandwidth, maximum stress, etc. However, the connecting beams of most microgrippers in the literature are based only on simple orthogonal structures^8,10,22–25^. Their shape is far from fully explored, and there is no evidence of achieving an optimal design. More complex, freeform geometrical shapes may result in a solution with superior performances, such as a much lower actuation voltage and a larger displacement. Therefore, we propose replacing simple orthogonal structures with structures based on freeform geometries and explore how this can improve the performance of the microgripper.

In our design methodology, Bezier curves were used to define and parameterize the freeform geometries in the connecting beam area. A curve can be described by a Bezier curve with only three coordinate points. Therefore, a beam can be defined by two Bezier curves, in which 12 parameters are used to describe the (*X*, *Y*) coordinates of 6 points, as shown in Fig. 2b. An orthogonal beam can be easily modified into a curved beam, as illustrated in Fig. 2b, in which only the coordinates of points P1 and P4 are modified. The number of parameters is significantly reduced, which saves computational resources for optimization.

### Parameter ranges and geometrical design constraints

The parameter ranges and geometrical design constraints were defined based on the fabrication process described in ref. ^26^ and are listed in Table 1. The minimum width of the freeform geometries was set as 7 µm to prevent parts from becoming too fragile. All parameterized variables have lower and upper bounds (LBs and UBs, respectively). LBs and UBs are determined either based on (i) practical limitations, such as fabrication tolerance and voltage limitation, or (ii) a qualified guess by the designer of the optimum value. It is important to clarify that the GA was used to optimize only the connected beam freeform geometry, gripper Arm geometry, rotary actuator length, and rotary actuator width. The GA algorithm was not applied to the other parts of the design that were related to generating electrostatic force among the comb fingers.

**Table 1:** Definition, symbol, and upper and lower bounds of parameters

Parameter	Symbol	LB	UB
Gripper arm length	*L*_*G*_	500 μm	1700 μm
Gripper arm width	*W*_*G*_	50 μm	150 μm
Arm tip length	*L*_*A*_	100 μm	200 μm
Arm tip width	*W*_*A*_	40 μm	100 μm
Arm tip angle	*A*_*A*_	35°	35°
Finger angle	*A*_*F*_	24°	24°
Finger angle offset	*O*_*F*_	4°	4°
Finger length	*L*_*F*_	44 μm	44 μm
Finger width	*W*_*F*_	5 μm	5 μm
Finger gap	*G*_*F*_	3 μm	3 μm
Rotary actuator length	*L*_*R*_	700 μm	1000 μm
Rotary actuator width	*W*_*R*_	15 μm	30 μm
Connecting beam length	*L*_*C*_	50 μm	300 μm
Connecting beam width	*W*_*C*_	7 μm	7 μm
Connecting beam top length	*L*_*CT*_	100	250
Connecting beam top width ratio	*WR*_*CT*_	0.5	0.5
Connecting beam top ShiftX	*SX*_*CT*_	−150 μm	150 μm
Connecting beam middle length ratio	*LR*_*CM*_	0.1	0.9
Connecting beam middle width ratio	*WR*_*CM*_	0.5	0.5
Connecting beam middle ShiftX	*SX*_*CM*_	−150 μm	150 μm
Connecting beam bottom width ratio	*WR*_*CB*_	0.5	0.5
Connecting beam bottom ShiftX	*SX*_*CB*_	−150 μm	150 μm

The movement of a rotary comb actuator after actuation can be best described by a polar coordinate system, as illustrated in Fig. 2c. The R-axis is defined along the radial direction of the rotary comb fingers; this is an undesired displacement direction of comb fingers and should be minimized^27^. The *θ*-axis is along the tangential direction of rotary comb fingers (perpendicular to the *R*-axis), which is the desired movement direction of comb fingers and should be maximized. The displacement of the rotary comb actuator along the *R*-axis, i.e., *R*_*m*_, reduces the gap between the fixed and moving combs. As a result, the gaps of a moving comb finger with respect to the two neighboring fixed comb fingers are no longer equal. With any further increase in the actuation voltage, electrostatic pull-in will thus occur if *R*_*m*_ is larger than one-third of the comb finger gap (4 μm), i.e., 1.3 μm^11^. The pull-in effect limits the maximum displacement of the microgripper. Therefore, one important constraint during the optimization process is that *R*_*m*_ needs to be less than 1.3 μm.

It is important to note that during the optimization, the design space for the connecting beams is fixed (390 × 390 μm^2^) for the GA; this enables objective comparison of different designs. It could be argued that for an orthogonal beam design, the actuation range can be improved by simply increasing the length of the connecting beam. However, in a fixed design space, the two adjacent orthogonal connecting beams will cross each other if the two connecting beams are prolonged beyond a certain level, which is obviously physically impossible. A serpentine orthogonal beam could be used to prevent this and prolong the beam length; however, this reduces the stiffness in the radial direction and thus increases *R*_*m*_, leading to a low pull-in event. Therefore, constraining the design to a conventional orthogonal shape does not fully explore the design space and does not achieve an optimal design. More complex freeform geometrical shapes may result in a solution with superior performance. Thus, we propose replacing simple orthogonal structures with structures based on freeform geometries. Their shapes can be optimized with the GA to improve the actuation range at a low actuation voltage.

### Figures of merit

In the following, we regard the sum of the displacements at the two gripper arm tips as the displacement of the microgripper, *X*_*T*_. Ideally, a large *X*_*T*_ with a low actuation voltage is desired for an electrostatically actuated microgripper. Therefore, *X*_*T*_ for a fixed actuation voltage (40 V) was used as the FOM for the design process. The gap between the arm tips of the microgripper was designed as 100 μm, which obviously defines an upper limit for *X*_*T*_. These values were chosen because most of the electrostatic microgrippers described in the literature require a voltage above 80 V to reach an *X*_*T*_ of 100 μm. Therefore, 40 V represents a typical mid-range actuation voltage, suitable for comparison.

Consequently, the GA is programmed in such a way that it maximizes *X*_*T*_ while maintaining *R*_*m*_ less than 1.3 μm.

### Optimization process

In the first step of the optimization process, the GA ran 40 individuals (i.e., designs with a specific parameter set), which were chosen randomly within the parameter ranges. For each individual, a FEM simulation was carried out for the fully parameterized mechanical model. The FEM simulation included a static displacement simulation for a fixed actuation voltage. For the simulation, the electromechanical multiphysics functionality in COMSOL was used, in which the electrostatic actuation force was calculated based on the number and geometry of the comb fingers and the actuation voltage. The value of *R*_*m*_ > 1.3 μm or a convergence failure of the simulation indicated a pull-in event. The simulation result was automatically transferred to the GA in MATLAB, which recorded and sorted the results based on the FOM and performed several postprocessing steps. These included picking the ten best individuals (elite preservation), deriving a certain number of new random individuals (mutation), and cross-fertilizing good individuals to create new offspring. This last step involved taking different parameters from different good individuals and combining them to create a new individual (child). These three steps created the parameter value set for the 2nd generation. Then, the GA started the same optimization process for the second generation as for the first generation. For each simulation, a row of values was recorded and displayed in the command window of MATLAB.

In the first generation, the FOM varied considerably, indicating that the algorithm still explored the design space. After the first generation, the GA already tended to find designs that have a large FOM. In the end, the GA consistently settled toward designs with a higher FOM and started to converge.

**Fig. 3:**
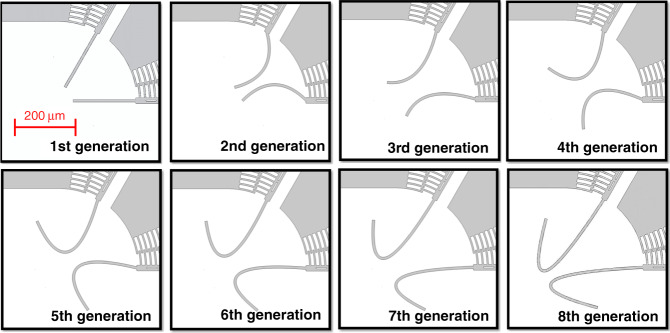
Optimization process. The shape of the connecting beams changes during the GA optimization

Figure 3 shows a graphical illustration of the optimization process, which went through eight generations. The GA considerably changed the shape of the connecting beams. During the optimization, the GA attempted to make the connecting beams more compliant by bending them to increase *X*_*T*_. In addition, the GA folded the connecting beams to increase their length, which further reduced the stiffness and improved *X*_*T*_. However, due to the rotary comb actuator, the connecting beams would not only move along the tangential axis but also exhibit undesired movement along the radial axis, as illustrated in Fig. 2c. This increased the displacement of the microgripper in the R-axis in Fig. 2c. Thus, the GA attempted to reduce *R*_*m*_ by making the bends of two curved connecting beams face each other. In that way, the undesired movement of two curved connecting beams was in opposing directions and canceled each other, reducing *R*_*m*_. Finally, the undesired movement of the rotary comb actuator was reduced. (this will be discussed in detail in Section V.C).

### Robustness analysis

The next step in the design process was a robustness analysis, which started by collecting 10 individuals with the highest FOM; these were taken as optimal design candidates.

For the robustness analysis, the designer had to specify a standard deviation of each design parameter representing the fabrication tolerances. One hundred Gaussian distributed parameter sets were calculated for all parameters of an individual using the mean value and designer-supplied standard deviations. These effectively represent the fabrication tolerances. Therefore, for each individual, 100 simulations were run, and the FOMs were recorded. A minimum threshold for the FOM was set by the designer. A yield value was calculated, representing the percentage of the simulations for each individual above the minimum FOM. The designer finally had to choose one as the final design by reviewing the yield and the FOM of the investigated individuals based on the application requirement.

## Optimization result

The GA optimization ran continuously for eight generations, with one generation size of 40 individuals, as shown in Fig. 3. As mentioned before, the minimum beam width for all designs was set to 7 μm during the optimization process. It is worth noting that the whole optimization process took 8 h with a 3D mechanical model and 6 h with a 2D mechanical model using a laptop with an i7 core of 2.5 GHz working frequency and 8 G RAM. The optimization was completed in 8 h, with little manual intervention. The optimization process would take much less time if it ran on a workstation or in a parallel computation mode.

Two types of freeform designs were selected as the optimal designs, referred to as CB7-D1 (Fig. 6b (1)) and CB7-D2 (Fig. 6b (2)); their parameter values are listed in Table 2. CB7-D1 had a larger *X*_*T*_ than CB7-D2. CB7-D2 had a larger *R*_*m*_ than CB7-D1. The difference between the two designs was mainly because CB7-D1 had a more compliant freeform beam than CB7-D2.

**Table 2:** Definition, symbol, and upper and lower bounds of parameters

Parameter	CB7-D1	CB7-D2	SB7
Gripper arm length	1520 μm	1534 μm	1545 μm
Gripper arm width	78 μm	88 μm	88 μm
Arm tip length	180 μm	210 μm	190 μm
Arm tip width	79 μm	85 μm	90 μm
Arm tip angle	35°	35°	35°
Finger angle	24°	24°	24°
Finger angle offset	4°	4°	4°
Finger length	44 μm	44 μm	44 μm
Finger width	5 μm	5 μm	5 μm
Finger gap	3 μm	3 μm	3 μm
Rotary actuator length	940 μm	949 μm	960 μm
Rotary actuator width	20 μm	18 μm	21 μm
Connecting beam length	230 μm	221 μm	200 μm
Connecting beam width	7 μm	7 μm	7 μm
Connecting beam top length	210	198	238
Connecting beam top width ratio	0.5	0.5	0.5
Connecting beam top ShiftX	5 μm	4 μm	0 μm
Connecting beam middle length ratio	0.6	0.7	0.5
Connecting beam middle width ratio	0.5	0.5	0.5
Connecting beam middle ShiftX	25 μm	30 μm	0 μm
Connecting beam bottom width ratio	0.5	0.5	0.5
Connecting beam bottom ShiftX	130 μm	112 μm	0 μm

To compare the freeform designs with a conventional orthogonal design, the same GA optimization algorithm was also run with constraints allowing only an orthogonal design. An identical design space (390 × 390 μm^2^) was chosen for the connecting beams to allow for an objective comparison. The optimal orthogonal design was termed SB7 (Fig. 6b (3)); Table 3 also lists its FOM. Compared with CB7-D1 and CB7-D2, SB7 had the lowest FOM. Here, 80% of the FOM value in each optimal design was taken as the minimum threshold of acceptable FOM values during the robustness analysis.

**Table 3:** The FOMs and simulated yield of microgripper design CB7-D1, CB7-D2, and SB7

Performance	FOM (μm)	Yield (%)
CB7-D1	59	80
CB7-D2	49	79
SB7	24	87

According to the robustness analysis, CB7-D1, CB7-D2, and SB7 had a yield of 86% (minimum FOM of 47 μm), 84% (minimum FOM of 39 μm), and 90% (minimum FOM of 19 μm), respectively.

As a freeform design has many degrees of freedom, it is necessary to disperse the parameter values during the optimization to achieve a global rather than a locally optimal solution. However, an excessively dispersed parameter space makes the optimization process computationally intensive. To study the convergence, the GA carried out ten independent optimization processes by using different initial designs across the design space. As circumstantial evidence, the topologies of ten optimal solutions resembled each other, indicating a global convergence of the optimization process to a large extent. The FOMs of the designs obtained in 10 different optimization runs ranged from 47 to 60 μm.

### Static analysis

**Fig. 4:**
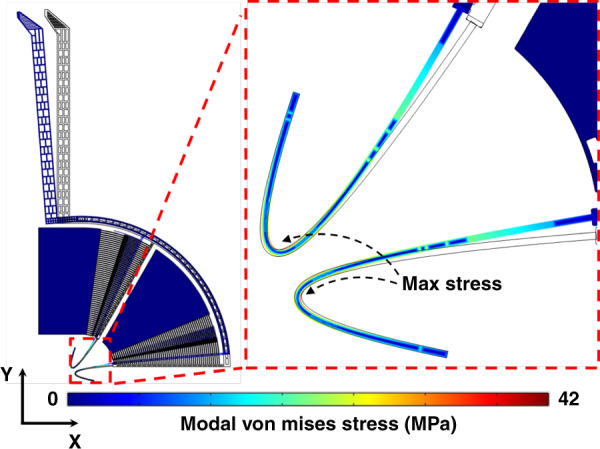
Optimization result. A von Mises stress contour plot of the optimal freeform design CB7-D1 with an actuation voltage of 53 V and an *X*_*T*_ of 100 μm

According to a FEM simulation in COMOL, the freeform design CB7-D1 had an *X*_*T*_ of 100 μm for a DC actuation voltage of 53 V, as shown in Fig. 4. The freeform design CB7-D2 had an *X*_*T*_ of 100 μm for a DC actuation voltage of 57 V. The optimized orthogonal design SB7 had an *X*_*T*_ of 41 μm for a DC actuation voltage of 53 V and 48 μm for a DC actuation voltage of 57 V. A DC actuation voltage of 85 V was required for the orthogonal design SB7 to reach an *X*_*T*_ of 100 μm. Comparing the freeform design CB7-D1 with the orthogonal design SB7, *X*_*T*_ was increased by 144% for the same DC actuation voltage of 53 V, as shown in Table 4. In addition, the actuation force for the freeform design CB7-D1 to reach an *X*_*T*_ of 100 μm was only 39% of that of the orthogonal design SB7, as shown in Table 4. Therefore, the stiffness of the connecting beams in the freeform design CB7-D1 is lower than that in the orthogonal design SB7. The output force of the gripper when grasping a micro-object is an important parameter of the gripper performance, which is directly related to the stiffness of the connecting beams. The freeform design CB7-D1 is thus expected to be less harmful to fragile samples during manipulation compared with the orthogonal design SB7.

**Table 4:** *X*_*T*_ of microgripper design CB7-D1, CB7-D2, and SB7 under different actuation voltages

DC actuation voltage (V)	53	57	85
CB7-D1 *X*_*T*_ (μm)	100	/	/
CB7-D2 *X*_*T*_ (μm)	83	100	/
SB7 *X*_*T*_ (μm)	41	48	100

### Dynamic analysis

Given the significant influence of vibration modes and stress on the microgripper, these parameters were analyzed next. The frequencies of the first three modes of freeform design CB7-D1 were 823, 10,583 and 27,932 Hz, respectively. The 2nd mode frequency is 11.86 times larger than the working mode (1st mode) frequency, which considerably increases the stability during actuation. The frequencies of the first three modes of freeform design CB7-D2 and orthogonal design SB7 are listed in Table 5; the mode shapes of CB7-D1 were very similar.

**Table 5:** First three modes of the microgripper design CB7-D1, CB7-D2, SB7

Design	1st Mode (Hz)	2nd Mode (Hz)	3rd Mode (Hz)
CB7-D1	823	10583	27932
CB7-D2	906	11484	29365
SB7	1245	16975	48530

### Stress analysis

In our design, the connecting beams are used to support the movable structures and to bend during a gripping operation. This makes the connecting beams the most fragile part of the design, and thus, they could break under a large electrostatic force input. Hence, a stress analysis was performed to predict the stress distribution of the microgripper during actuation. According to a FEM simulation in COMSOL, when the freeform microgripper CB7-D1 reached 100 μm (its maximum *X*_*T*_), the maximum Von Mises stress was 42 MPa (as shown in Fig. 4), which is much smaller than the yield strength of single-crystal silicon, i.e., 7 GPa^28^. This low-stress value is another benefit of the freeform geometries and the GA optimization. Compared with orthogonal beams, stress can be more evenly distributed by the curved shapes of freeform beams, and stress concentration can be prevented. Additionally, the GA attempted to reduce the stress to increase the X_T_ since a low-stress concentration leads to a large *X*_*T*_. As shown in Fig. 4, the stress was evenly distributed on the freeform. As will be discussed later, in the experiment, none of the microgrippers broke during actuation. Furthermore, the microgripper did not break even when we manually probed the arm tips of the microgripper to release them from the actuation combs after a pull-in event. As shown in Fig. 4, the maximum Von Mises stress of CB7-D1 was located at the turning point of the freeform beam. The maximum Von Mises stresses of microgripper design CB7-D2 and design SB7 were 44 and 179 MPa, respectively, when they reached an *X*_*T*_ of 100 μm.

## Fabrication

**Fig. 5:**
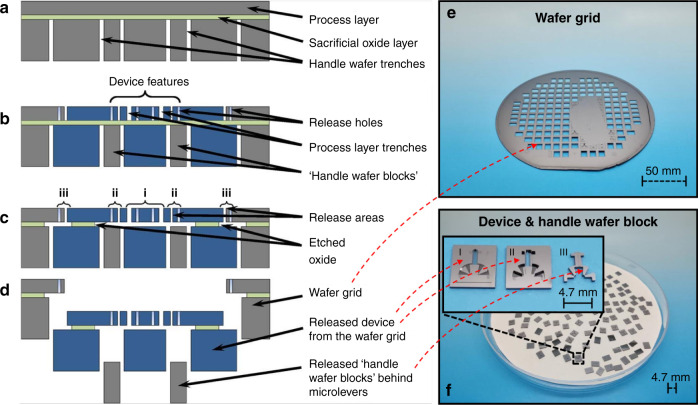
Fabrication process. Fabrication flow of the MEMS devices: **a** Backside etching using DRIE to define the backside trenches. **b** Front side DRIE to pattern the device features, release holes, and front side trenches. **c** Three release regions, namely, (i) device, (ii) handle wafer block release features, and (iii) dicing features, were etched consecutively by hydrofluoric acid in the vapor phase. **d** Device separation after release^26^. **e** Image of the wafer grid of step (**f**) (the solid area resulting from a lithography fault). **f** Image of the released devices. (I) The front image of the released device, (II) back image of the released device, and (III) released “handle wafer blocks”

Figure 5 shows the SOI-based process flow used in this work, which is similar to that described in ref. ^26^. After etching a pattern of frame trenches on the handle layer of a wafer by deep reactive-ion etching, another pattern of trenches and etch holes were etched on the front side in a 50-μm-thick device layer. The handle layers beneath the rotary comb actuators, gripper arms, and arm tips were removed to increase yield and reliability by offsetting the two trench patterns by 40 µm. Finally, the devices were separated from each other by HF vapor phase etching without the usage of a dicing step.

**Fig. 6:**
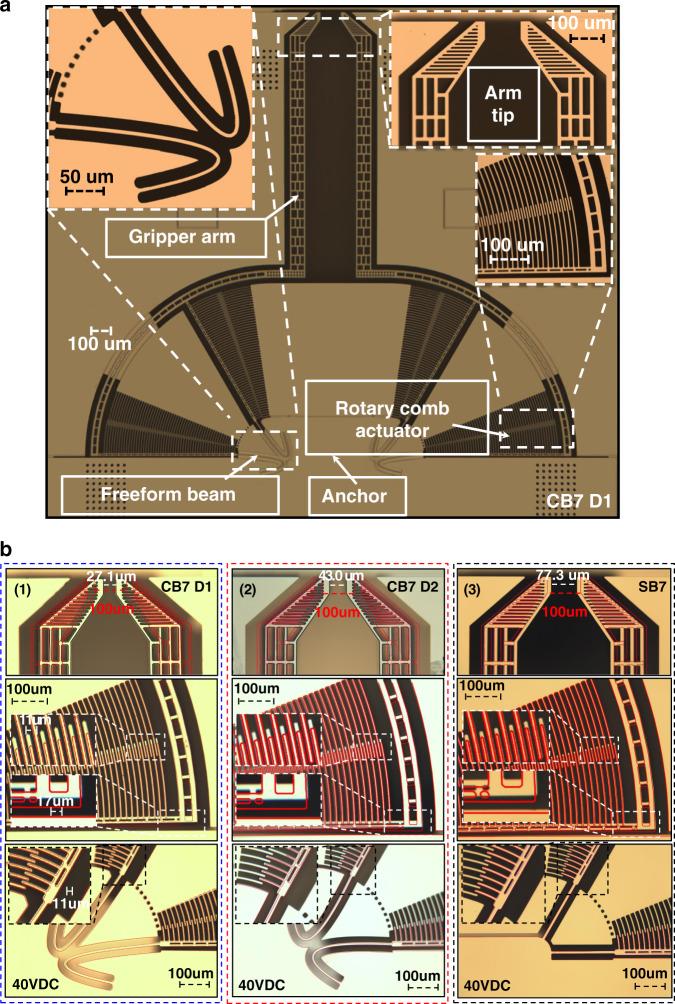
Fabricated microgrippers and their displacement under 40 V actuation. **a** Metallographic microscope image of the freeform microgripper CB7-D1. **b** The images of the microgripper CB7-D1, CB7-D2, and SB7 under a certain actuation voltage. Image (1)–(3). The images of the microgripper CB7-D1, CB7-D2, and SB7 under an actuation voltage of 40 V. For comparison, the red contours indicate the position of the structure before actuation. The upper images show the arm tip area, the middle shows the rotary comb actuator area, and the bottom images show the connecting beam area. (1) CB7-D1, (2) CB7-D1, and (3) SB7 for an actuation voltage of 40 V. For comparison, the red contours indicate the position of the structure before actuation. The upper images show the arm tip area. The middle images show the rotary comb actuator area. The bottom images show the connecting beam area

Figure 6a shows the fabricated microgripper CB7-D1 with the curved shapes of the freeform beams. For designs CB7-D2 and SB7, the structure was identical to CB7-D1, except for the connecting beams. A comparison of the connecting beams of CB7-D1, CB7-D2, and SB7 is shown in Fig. 6b. We fabricated 172 chips on a 4-in. wafer, including freeform and orthogonal designs with a chip size of 3.7 × 3.7 mm^2^. Approximately, 90–95% of all fabricated chips had complete structures and were fully functional after release, bonding, and packaging. This fabrication result indicated that the yield rate of the freeform MEMS devices was as good as that of the orthogonal MEMS designs as long as rules concerning minimum feature size (such as minimum etching trenches, minimum widths) were followed.

## Experiment results and discussion

### Experiment setup

**Fig. 7:**
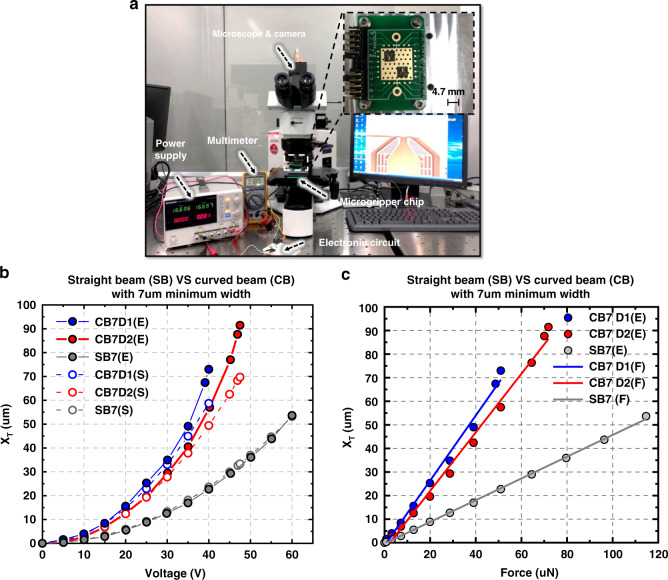
Measurement setup and result. **a** Measurement setup. **b** Characterization of the simulated and measured X_T_ versus actuation voltages in two freeform designs (CB7-D1 (blue line), CB7-D2 (red line)) and one orthogonal design (SB (black line)). Simulated results (dashed lines): CB-7D1(S), CB7-D2(S), SB7(S). Measured results (solid lines): CB7-D1 (E), CB7-D2 (E), SB7 (E). **c** Characterization of the simulated and measured *X*_*T*_ vs. actuation force in two freeform designs (CB7-D1 (blue), CB7-D2 (red)) and one orthogonal design (SB (black)). Linear fitting lines (solid lines): CB-7D1 (F), CB7-D2 (F), SB7 (F). Measured results (dots): CB7-D1(E), CB7-D2(E), SB7(E)

As shown in Fig. 7a, the measurement setup included a voltage source, a multimeter, a microscope with a camera, and an electronic circuit. The voltage source could supply a DC voltage ranging from 0 to 60 V. The multimeter was used to measure the exact voltage supplied to the microgripper. A microscope with a camera was used to measure the displacement and gripping action of the microgripper. The electronic circuit included some protecting resistors in case pull-in occurred and the current would become too high.

### Gripping range test result

First, the gripping ranges were tested. Two types of freeform designs, i.e., CB7-D1 (blue line) and CB7-D2 (red line)), and one orthogonal design, i.e., SB (black line), were tested. When different voltages were applied to the microgrippers, the images of the arm tips were acquired and processed to calculate the displacement. In Fig. 7b, the experimental results of three types of microgrippers are shown with a solid line, i.e., CB7-D1(E), CB7-D2(E), and SB7(E). The experimental results indicated that the microgripper design CB7-D1 provided a gripping range of 73 μm with an actuation voltage of 40 V and design CB7-D2 gripping range of 91.5 μm with an actuation voltage of 47.5 V. Limited by the maximum voltage of the voltage source, microgripper design SB7 provided a gripping range of 48.0 μm with an actuation voltage of 60 V. Since the orthogonal design SB7 is only used to evaluate the improvement of the freeform designs CB7-D1 and CB7-D2 under the same actuation voltage, 60 V was sufficient for testing design SB7.

In Fig. 7b, the simulated results of the respective designs are also plotted (dashed lines), i.e., CB7-D1(S), CB7-D2(S), and SB7(S). The experimental results agree well with the simulation results. The small discrepancy is due to fabrication tolerances of the gripper parameters and the pull-in effect.

The displacements of the microgrippers were compared not only at the arm tips but also in the areas of the rotary comb actuators and connecting beams. A comparison of the three types of microgripper designs with an actuation voltage of 40 V is shown in Fig. 6b (1)–(3), in which the red contours indicate the position of the structure before actuation. The X_T_ of design CB7-D1 was larger than that of design CB7-D2, which, in turn, was larger than that of design SB7 in all three areas. Since CB7-D1 could not be actuated higher than 40 V (which is close to the pull-in voltage), the comparison of designs CB7-D2 and SB7 was made with an actuation voltage of 47.5 V. The *X*_*T*_ of design CB7-D2 was much larger than that of the design SB7 in all three comparison areas. In summary, for the same actuation voltage, microgrippers with freeform geometries improved the *X*_*T*_ by 150–200% compared with orthogonal geometries in the same die area.

Figure 7c shows the relationships between the actuation force and *X*_*T*_ for the three designs, i.e., CB7-D1, CB7-D2, and SB7. Linear fittings were plotted using the least-squares method. In terms of the connecting beam stiffness, CB7-D1 has a nonlinearity of 5.5% in the worst case for a 51 μN actuation force range; CB7-D2 has a nonlinearity of 5.6% in the worst case for a 72 μN actuation force range; SB7 has a nonlinearity of 2.2% in the worst case for a 115 μN actuation force range. CB7-D1, CB7-D2, and SB7 have a nonlinearity of 5.5%, 5.2%, and 1.2% in the worst case for a 51 μN actuation force range, respectively. As shown in Fig. 7c, among the three designs, SB7 has the lowest nonlinearity of the connection beam stiffness under the same actuation force range, as SB7 has the highest stiffness (the smallest *X*_*T*_ under the same actuation force). CB7-D2 and CB7-D1 have the second and third lowest nonlinearities of the connection beam stiffness under the same actuation force. Thus, the higher the connecting beam stiffness is, the lower the nonlinearity of the connecting beam stiffness under the same actuation force is.

According to the simulation, the total capacitance of the rotary comb actuators in CB7-D1 changes from 2.28 to 3.16 pF after achieving a deflection *X*_*T*_ of 72.9 μm. The total capacitance of the rotary comb actuators in CB7-D2 changes from 2.28 to 3.28 pF after achieving a deflection *X*_*T*_ of 91.5 μm. The total capacitance of the rotary comb actuators in S7-D2 changes from 2.28 to 3.00 pF after achieving a deflection *X*_*T*_ of 54 μm. The effect of the fringing field does not play an important role and can be ignored during the actuation process^8,10^.

### Pull-in of rotary comb drives

**Fig. 8:**
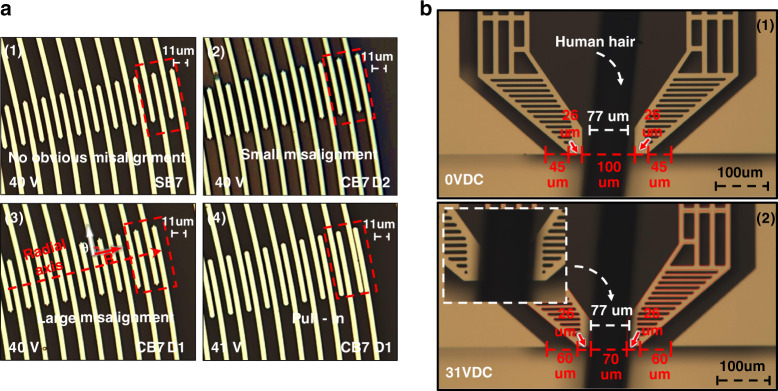
Results of 47.5 V actuation experiment, pull-in experiment, and gripping experiment. **a** Images of designs CB7-D2 and SB7 under an actuation voltage of 47.5 V. Comparison of radial deflection *R*_*m*_ of the rotary comb actuators for different microgripper designs. (1) No apparent *R*_*m*_ in the microgripper design SB7 under an actuation voltage of 40 V. (2) Small *R*_*m*_ in the microgripper design CB7-D2 under an actuation voltage of 40 V. (3) Large *R*_*m*_ in the microgripper design CB7-D1 under an actuation voltage of 40 V. (4) Pull-in of the microgripper design CB7-D1 under an actuation voltage of 41 V. **b** Gripping a human hair with a diameter of 77 μm using microgripper design CB7-D2 (1) before gripping and (2) after gripping

For design CB7-D1, an actuation voltage higher than 40 V led to the pull-in of the rotary comb actuators, as shown in Fig. 8a. For an actuation voltage of 40 V, the *R*_*m*_ of SB7 was not observable, whereas the *R*_*m*_ of CB7-D2 was approximately two times smaller than that of CB7-D1. In addition, pull-in occurred when the gripper of design CB7-D1 moved 74 μm under an actuation voltage of 41 V. Pull-in occurred in design CB7-D1 due to *R*_*m*_ becoming too large, resulting from the undesired movement of the curved beam along the *R*-axis. As shown in Fig. 8a. (4), the outermost comb fingers had the largest *R*_*m*_ value compared with other comb fingers and reached the pull-in point first, as the long lever of the rotary comb actuator acts as a motion amplifier.

The pull-in effect can easily be mitigated by increasing the stiffness of the connecting beams along the *R*-axis (e.g., by increasing the beam width). However, this will reduce the *X*_*T*_ for a given actuation voltage. After optimization, design CB7-D2 reached a larger *X*_*T*_ (91.5 μm) with a higher pull-in voltage (47.5 V).

### Demonstration of micro-object gripping

To demonstrate the performance of the fabricated microgripper, microgripper design CB7-D2 was used to grip human hair with a diameter of 77 μm. The position of the microgripper relative to the hair before the gripping test is shown in Fig. 8b (1), in which the gap of the arm tips is 100 μm. It is worth noting that the micro stick-slip motion between the object and arm tip is mainly determined by the friction force. In ref. ^29^, Zhang and Liu et al. found that micro stick-slip motion could be explained by the Stribeck model, Dahl model, and LuGre model. The LuGre model has the best accuracy. The Coulomb friction model and the elastoplastic model do not work in a micro stick-slip motion system.

Then, the microgripper was driven with a voltage of 31 V and gripped the hair, as shown in Fig. 8b (2). The measured gap of the arm tips was 70 μm, smaller than the diameter of the hair, indicating successful gripping of the hair. In addition, according to Fig. 7b, CB7-D2 was expected to have an *X*_*T*_ of 30 μm for an actuation voltage of 31 V, matching the experimental result shown in Fig. 8b (2).

## Discussion

For the same actuation voltage, microgrippers with freeform geometries (CB7-D1 and CB7-D2) improved *X*_*T*_ by 150–200% compared with orthogonal geometries (SB7) for the same die area. Therefore, the use of freeform geometries has two practical advantages: (i) a lower actuation voltage to reach the same *X*_*T*_ and (ii) less harm to fragile objects during gripping and releasing.

However, electrostatic rotary microgrippers exhibit an undesired radial displacement *R*_*m*_ during actuation. This leads to a reduction in the gap of the comb drive electrode, potentially causing pull-in, which limits the maximal *X*_*T*_. With the proposed optimization method, the *R*_*m*_ of the rotary comb actuators is included in the FOM. The GA-based optimization concurrently maximizes *X*_*T*_ and minimizes *R*_*m*_ for a given voltage.

Comparing the two freeform designs, CB7-D1 has a larger *X*_*T*_ but a larger *R*_*m*_ compared with CB7-D2 for the same actuation voltage. Thus, a designer can select freeform designs according to different requirements for the gripping range. For example, for objects with a diameter between 100 and 30 μm, design CB7-D1 is superior to CB7-D2, as CB7-D1 can satisfy the gripping range with a lower actuation voltage. Additionally, for objects with a diameter between 100 and 10 μm, CB7-D2 is better than CB7-D1, as CB7-D1 can offer a larger gripping range, while CB7-D1 pulls in after 74 μm.

Table 6 compares the gripping range of our microgrippers with those of other electrostatically actuated microgrippers in the literature. To compare the actuation ability of different designs fairly, the maximum *X*_*T*_ is divided by the square of the related actuation voltage, and the calculated result is taken as the actuation ability. Crescenzi et al.’s design^9^ have the highest actuation ability and lowest actuation force but a limited gripping range. Compared with Crescenzi et al.’s design, the CB7-D1 actuation ability is 6 times lower, while the gripping range is 3.6 times larger, whereas the design CB7-D2 actuation ability is 5 times lower, while the gripping range is 4.6 times larger.

**Table 6:** Comparison of different gripper operating displacements

Design	Actuation force (μN)	Actuation voltage (V)	Actuation range (μm)	Actuation ability (nm/V^2^)	Die area (μm^2^)	Gripper arm length (mm)
Volland^30^	231	80	20	3.13	1250*3300	1
Beyeler^22^	986	150	100	4.44	7700*5600	3.3
Chen^23^	1646	80	25	3.91	5745*3217	1
Bazaz^25^	1181	50	17	6.80	4891*6402	2.5
Chang^10^	297	100	94	9.40	3100*3700	1.7
Piriyanont^31^	104	80	90	14.06	8500*5600	1.6
Xu^32^	273	72	63	12.15	2800*3812	NA
Hao^8^	37	31.5	100	100.78	4500*4000	1.7
Crescenzi^9^	3	11	20	165.29	2710*4417	1.4
(CB7-D1)	51^a^	40	72.9	45.54	3700*3700	1.7
(CB7-D2)	72^a^	47.5	91.5	40.55	3700*3700	1.7
(SB7)	115	60	54	15.00	3700*3700	1.7

^a^From COMSOL simulation.

Hao et al.’s design^8^ has the second-highest actuation ability and largest gripping range. The high actuation ability in Hao et al.’s design is due to its narrow beamwidth (3.6 μm). If the beamwidth of CB7-D2 was reduced from 7 to 3.6 μm, a simulation indicated that CB7-D2 would only need 27 V to have an *X*_*T*_ of 100 μm, which is smaller than the 31 V of Hao et al.s design. Moreover, the actuation voltage can be further reduced through an increase in the number of rotary comb actuators, since Hao et al.’s design has six groups of rotary comb actuators, while our designs only have four groups of rotary comb actuators.

In addition, the CB7-D1 and the CB7-D2 design were developed based on Chang et al.’s design^10^. Compared with Chang et al.’s design^10^, the CB7-D1 actuation ability is 4.8 times larger, while the gripping range is 1.4 times lower, whereas the design CB7-D2 actuation ability is 4.3 times larger, while the gripping range is 1.1 times lower.

## Conclusions

A novel microgripper with freeform geometries designed using a GA approach is presented. The GA-based semiautomated design methodology with freeform geometries is introduced in detail. It is capable of designing near-optimal MEMS devices that are robust to fabrication tolerances. Two types of microgrippers with freeform geometries and one microgripper with orthogonal geometries were optimized by this method. FEA simulations were used to analyze the static and dynamic performance as well as the stress distribution of the designed microgrippers. The experiment showed that the microgripper with freeform geometries had a large *X*_*T*_ (91.5 μm) for a low actuation voltage (47.5 V), which agreed well with the theory. This made it possible to manipulate a wide range of objects (size ranging from 10 to 100 μm). The concept was successfully demonstrated by grasping a human hair with a diameter of 77 μm. A detailed analysis of the pull-in effect due to the *R*_*m*_ of the actuator electrodes was conducted. Possible methods to mitigate this effect were also discussed.

For the same actuation voltage, microgrippers with freeform geometries improved *X*_*T*_ by 150–200% compared with orthogonal geometries in the same die area. Thus, freeform geometries have two advantages: (i) a lower actuation power to reach the same *X*_*T*_ and (ii) less harm to fragile objects during gripping and releasing.

In Table 6, we briefly compare our freeform geometry design with the two best electrostatic microgrippers described in the literature^8,9^ in terms of actuation range and *X*_*T*_ per voltage 2 (actuation ability). Both freeform geometries developed in this work have a larger gripping range compared to Crescenzi et al.^9^. If the same number of actuation comb fingers is considered, our designs have a better actuation ability compared to Hao et al.^8^.

The improved performance of the microgripper is mainly due to the use of GA for freeform geometric design. It is worth noting that the proposed design methodology enabling freeform geometries can be extended to a wide range of other MEMS devices. Future work will include equipping the microgripper with both force sensing and a feedback system. This will allow the gripping process to be performed with higher precision and more controllable force, creating the ability for fast, automated operation.

## Supplementary information

Revised manuscript in PDF format
