# Supplementary material for: Design of a large-range rotary microgripper with freeform geometries using a genetic algorithm
Source: Microsyst Nanoeng. 2022 Jan 6;8:3. doi: 10.1038/s41378-021-00336-0 (PMC8733005; doi:10.1038/s41378-021-00336-0)
Supplement: Supplementary file 1 — Revised manuscript in PDF format [file 41378_2021_336_MOESM1_ESM.pdf]

Design of a Large-Range Rotary Microgripper with Freeform Geometries using a Genetic Algorithm

Chen Wang[1,2,3], Yuan Wang[4], Weidong Fang[1], Xiaoxiao Song[4], Aojie Quan[3], Michiel Gidts[3], Hemin Zhang[3], Huafeng Liu[4,*], Jian Bai[1,*], Sina Sadeghpour[3], and Michael Kraft[3]

[1] College of Optical Science and Engineering, Zhejiang University, China;
[2] Department of Electrical Engineering and Computer Science, University of Liege, Belgium;
[3] ESAT-MICAS, University of Leuven, Belgium;
[4] PGMF and School of Physics, Huazhong University of Science and Technology, China;
* Correspondence: huafengliu@hust.edu.cn (Tel : 86-027-87543880; Fax : 86-027-87556576); bai@zju.edu.cn (Tel : 86-0571-87951186; Fax : 86-0571-87951186)

Email of authors: Chen Wang (chenwang767@163.com); Yuan Wang (yuan.wang@uliege.be); Weidong Fang (fangwd@zju.edu.cn); Xiaoxiao Song (songxx@hust.edu.cn); Aojie Quan (aojie.quan@student.kuleuven.be); Sina Sadeghpour (sina.sadeghpourshamsabadi@kuleuven.be); Michiel Gidts (michiel.gidts@esat.kuleuven.be); Hemin Zhang (hemin.zhang@kuleuven.be); Jian Bai (bai@zju.edu.cn); Huafeng Liu (huafengliu@hust.edu.cn); Michael Kraft (michael.kraft@kuleuven.be).

Abstract: This paper describes a novel electrostatically actuated microgripper with freeform geometries designed by a genetic algorithm. The novel, semi-automated design methodology is capable of designing near-optimal MEMS devices that are robust to fabrication tolerances. The use of freeform geometries designed by a genetic algorithm significantly improves the performance of the microgripper. The experiment shows that the designed microgripper has a large displacement (i.e., 91.5 μm) with a low actuation voltage (47.5 V), which agrees well with the theory. The microgripper has a large actuation displacement and can handle micro-objects with a size from 10 μm to 100 μm. A grasping experiment on human hair with a diameter of 77 μm was performed to prove the functionality of the gripper. The result confirmed the superior performance of the new design methodology enabling freeform geometries. This design method can also be extended to the design of many other MEMS devices.

Keywords: Micro-electro-mechanical systems (MEMS), microgripper, freeform geometries, genetic algorithm (GA), large range, compliant mechanism, optimization.

1. Introduction

Microelectromechanical systems (MEMS) microgrippers are micro-scale grippers fabricated through a micromachined process, which consist of actuators, mechanical parts to perform the handling and manipulation of micro-objects (1-100 μm), as well as force sensors. MEMS microgrippers are widely used in handling cells and tissues in biology [1], micro assembly and testing mechanical property of micromachined devices [2].

MEMS microgrippers of different shapes, actuation as well as sensing principles have been developed in recent years. Designs reported in [3,4] are thermally actuated microgrippers. These microgrippers have a large displacement and a low actuation voltage. However, the high working temperature of thermally actuated microgrippers can be harmful to living cells and tissues in biological manipulation. Another design described in [5] is based on a piezoelectric actuated microgripper. Although this design features a large displacement and a large bandwidth, it requires a complicated fabrication process and exhibits hysteresis nonlinearity, which severely limits its spatial resolution during manipulation.

Moreover, piezoelectric actuated microgrippers cannot work in a high temperature environment. A magnetically actuated gripper was reported in [6]. This design provides a large displacement and a quick response with a reasonable sensitivity, but it requires a complicated and expensive assembly process. Alternatively, a microgripper based on shape memory alloy was discussed in [7]. This design had excellent flexibility and a large bandwidth. However, it also suffered hysteresis nonlinearity and a large power consumption. Electrostatically actuated microgrippers were reported in [8,9]. Especially, for the first time, Chang *et al*. introduced a rotary actuation comb into an electrostatically actuated microgripper to increase the displacement range to 94 μm with an actuation voltage of 100 V [10]. These designs feature a fast response time, low power consumption as well as no hysteresis. However, these designs have a relatively large dimension due to the high number of actuation comb fingers required. Besides, the maximum displacement of the electrostatically actuated microgripper is limited by the pull-in effect [11]. Also, the actuation voltage of the electrostatically actuated microgrippers is relatively high and normally, a voltage larger than 80 V is required to achieve a displacement of 100 μm. Such a high actuation voltage is not only problematic in practical applications but also can damage gripped samples.

In the vast majority of MEMS devices, simple geometrical layouts comprising only a few simple building blocks, such as beams, rectangular masses and, more rarely, rings or disk-shaped structures are used [12]. As discussed in the following, there are cases in which such conventional, simple designs limit the performance of MEMS devices and therefore may not meet the requirements for specific applications. Compared with conventional designs, geometries comprising more complex geometries offer the designer more freedom. Complex geometries may result in novel designs with superior performance [13] and overcome the limitation of simple mechanisms[14-17]. For example, by

using curved anti-springs, Middlemiss *et al.* [14] and Boom *et al.* [15] developed MEMS accelerometers with resolutions of nano-g level. These anti-springs features a low effective spring constant that cannot be achieved with conventional orthogonal designs, under the same fabrication constraints. However, complex theoretical calculations are needed to design such complex geometries. Such a design method requires designers to have considerable expertise and is practically impossible to transfer to other devices; a case-by-case approach is required. An alternative is topology optimization, which can be used to design MEMS devices with complex geometries. Ananthasuresh *et al.* [16,17] and Seshia *et al.* [18] developed complex force and motion amplification mechanisms to increase the sensitivity of accelerometers. Cao and Zhang *et al.* developed a module optimization method as a unified design approach for both compliant mechanisms and rigid-body mechanisms [19]. In the module optimization approach, the states of joints and links are fully parameterized, with which a designer can obtain a rigid-body mechanism, a partially compliant mechanism, or a fully compliant mechanism for a given design objective. However, in these MEMS devices, typically simple beam (or truss) elements are used as a fundamental building block to form optimized topologies. Such methodology easily results in designs that often cannot be fabricated, since it is difficult to implement fabrication constraints well in the topology optimization process [18].

In this paper, we introduce a novel electrostatically actuated microgripper with freeform geometries designed by a genetic algorithm (GA) approach. The novel design approach is introduced by describing the optimization process for a microgripper as a case study. In our previous work, GA was introduced for the first time for the design of freeform geometries for MEMS sensors. Specifically, a MEMS accelerometer comprising a mechanical motion amplifier was described to demonstrate the effectiveness of the design approach [20]. In the following, we describe a MEMS actuator (i.e., microgripper) with freeform geometries that were designed and optimized by the GA based design method. Due to the freeform geometries, the designed microgripper features a large displacement with a low actuation voltage compared with previously described electrostatic microgrippers. Detailed theoretical analysis and experimental validation were conducted. A manipulation experiment using the designed microgripper for grasping human hair is shown. Moreover, the pull-in effect in the electrostatically actuated microgrippers is also discussed. The performances of the designed microgrippers are compared with those of existing micro-grippers.

2. Design of the Microgripper with Freeform Geometries

2.1 Design Methodology based on a Genetic Algorithm

The microgripper in this work was designed using a novel design method allowing freeform geometries based on a GA. The methodology comprises two parts, a parametrized mechanical finite element model (FEM) with freeform geometries implemented in COMSOL [21]and a GA implemented in MATLAB [21], illustrated by the flow-chart in Fig. 1. The FEM model and simulation in COMSOL can be directly controlled by the MATLAB through LiveLink for MATLAB [21]. A GA is based on the mechanics of natural selection and genetics, combining the fittest individuals in the population in order to search for the best solution. These evolutionary based techniques are excellent for particularly complex, multi- parameter problems for which they are capable of finding good solutions in a short period of time. For optimization, the GA sets the parameter values of a mechanical model and simulates each "individual" parameter set in the 1st generation. Using a performance goal (or figure of merit (FOM)) function, the GA generates a new parameter set for the next generation. After several generations, the parameter values converge, indicating the mechanical model reaches an optimal design. The details are the design process are described in the following.

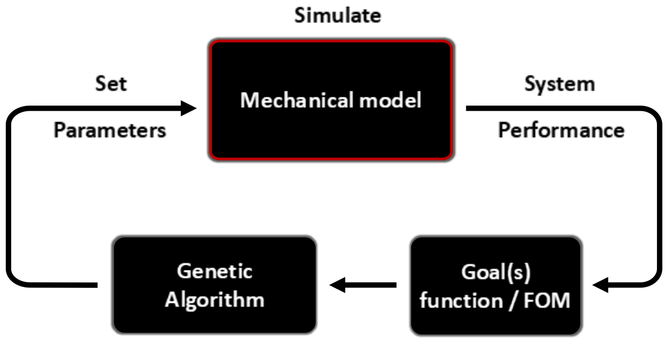

Fig. 1 Optimization system. Generic process flow of the novel designed method with freeform geometries based on a GA.

2.2 Microgripper Model with Freeform Geometries

A schematic drawing of a microgripper with freeform geometries is shown in Fig. 2a It comprises rotary comb actuators, two gripper arms, two arm tips to grasp micro-objects as well as connecting beams that link the moveable structures to anchors. The gap between the two arm tips is 100 μm. When a voltage is applied to the rotary comb actuators, due to electrostatic force, the microgripper will move in the direction of the blue arrows, as indicated in Fig. 2a. This displacement is mechanically amplified and transferred to the arm tips through the gripper arms; this effectively functions like a mechanical lever [10]. The critical part of the microgripper is the connecting beam. It defines

the total stiffness of the structure, which influences actuation voltage, actuation displacement, bandwidth, maximum stress, etc. However, the connecting beams of most microgrippers in the literature are based only on simple orthogonal structures [8,10,22-25]. Their shape is far from fully explored, and neither there is evidence of achieving an optimal design. More complex, freeform geometrical shapes may result in a solution with superior performances, such as a much lower actuation voltage and a larger displacement. Thus, we propose to replace the simple orthogonal structures with structures based on freeform geometries and explore how this can improve the performance of the microgripper.

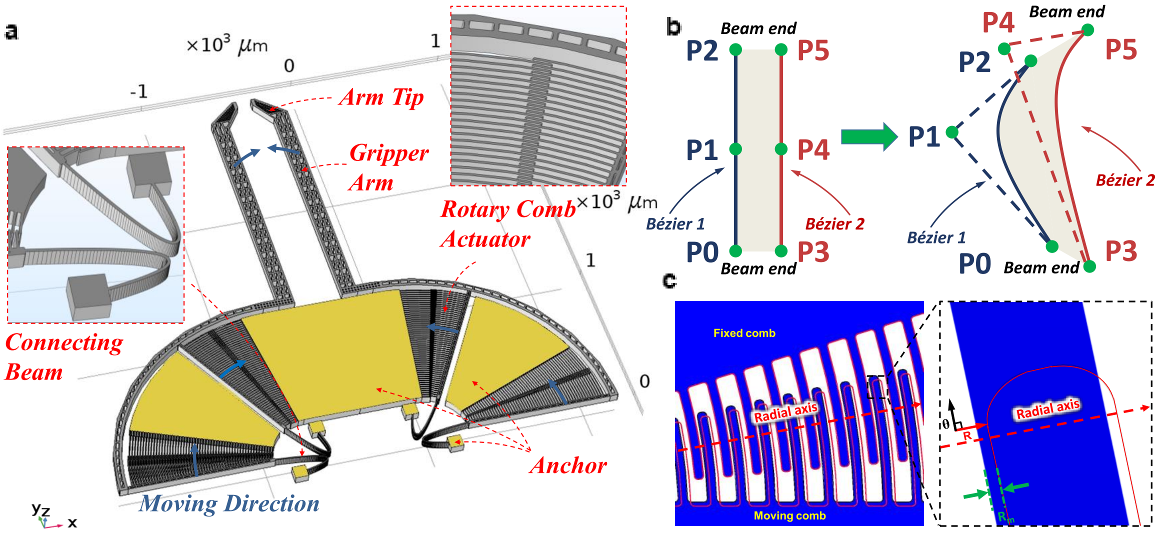

Fig. 2 Proposed microgripper with freeform geometries and misalignment of rotary microgrippers. a Schematic view of the proposed microgripper with freeform geometries showing arm tip, gripper arm, rotary comb actuator, connecting beam (freeform geometries), anchor. The moving direction of the microgripper is along the direction of blue arrows. **b** With the usage of Bezier curves, any beam can be defined with the coordinates of 6 points. An orthogonal beam can be modified into a curved beam easily by just modifying the coordinates of the P1 and P4. **c** Movement of a rotary comb actuator after actuation. The red line is the position of the moving comb before actuation, while the solid blue part is the position of the moving comb after actuation. The R-axis is along the radial axis of rotary comb fingers, which is the undesired displacement of comb fingers. The θ-axis is along the tangent direction of rotary comb fingers (perpendicular to the R-axis), which is the desired movement direction of comb fingers. The undesired movement along the radial axis, i.e., R_m, reduces the gap between the fixed and moving comb fingers.

In our design methodology, Bezier curves were used to define and parameterize the freeform geometries in the connecting beam area. A curve can be described by a Bezier curve with only three coordinate points. Therefore, a beam can be defined by two Bezier curves, in which 12 parameters are used to describe the (X, Y) coordinates of 6 points, as shown in Fig. 2b. An orthogonal beam can be modified into a curved beam easily as illustrated in Fig. 2b, in which only the coordinates of points P1 and P4 are modified. The number of parameters is significantly reduced, which saves computation resources for the optimization.

2.3 Parameter ranges and geometrical design constraints

The parameter ranges and geometrical design constraints were defined based on the fabrication process described in [26] and are listed in Table 1. The minimum width of the freeform geometries was set as 7 µm to prevent parts from becoming too fragile. All parameterized variables have lower and upper bounds (LBs and UBs, respectively). LBs and UBs are determined either based on (i) practical limitations, such as fabrication tolerance, voltage limitation, etc, or (ii) a qualified guess by the designer of the optimum value. It is important to clarify that the GA was used to optimize only the connected beam freeform geometry, gripper Arm geometry, rotary actuator length, rotary actuator width. The GA algorithm was not applied to the other parts of the design that were related to generate electrostatic force among the comb fingers.

Table 1 definition, symbol, and upper and lower bounds of parameters

Parameter	Symbol	LB	UB
GripperArmLength	L_G	500 µm	1700 µm
GripperArmWidth	W_G	50 µm	150 µm
ArmTipLength	L_A	100 µm	200 µm
ArmTipWidth	W_A	40 µm	100 µm
ArmTipAngle	A_A	35 °	35 °
FingerAngle	A_F	24 °	24 °
FingerAngleOffset	O_F	4 °	4 °
FingerLength	L_F	44 µm	44 µm
FingerWidth	W_F	5 µm	5 µm
FingerGap	G_F	3 µm	3 µm
RotatryActuatorLength	L_R	700 µm	1000 µm
RotatryActuatorWidth	W_R	15 µm	30 µm
ConnetingBeamLength	L_C	50 µm	300 µm
ConnetingBeamWidth	W_C	7 µm	7 µm
ConnetingBeamTopLength	L_{CT}	100	250
ConnetingBeamTopWidthRatio	WR_{CT}	0.5	0.5
ConnetingBeamTopShiftX	SX_{CT}	-150 µm	150 µm
ConnetingBeamMiddleLengthRatio	LR_{CM}	0.1	0.9
ConnetingBeamMiddleWidthRatio	WR_{CM}	0.5	0.5
ConnetingBeamMiddleShiftX	SX_{CM}	-150 µm	150 µm
ConnetingBeamBottomWidthRatio	WR_{CB}	0.5	0.5
ConnetingBeamBottomShiftX	SX_{CB}	-150 µm	150 µm

The movement of a rotary comb actuator after actuation can be best described by a polar coordinate system, as illustrated in Fig. 2c. The R-axis is defined along the radial direction of the rotary comb fingers; this is an undesired displacement direction of comb fingers and should be minimized [27]. The θ-axis is along the tangential direction of rotary comb fingers (perpendicular to the R-axis), this is the desired movement direction of comb fingers and should be maximized. The displacement of the rotary comb actuator along the R-axis, i.e., R_m, reduces the gap between the fixed and moving combs. As a result, the gaps of a moving comb finger with respect to the two neighbouring fixed comb fingers are not equal anymore. With any further increase of the actuation voltage, electrostatic pull-in will thus occur, if R_m is larger than one-third of the comb finger gap (4 µm), i.e., 1.3 µm [11]. The pull-in effect limits the maximum displacement of the microgripper. Therefore, one important constraint during the optimization process is that R_m needs to be less than 1.3 µm.

It is important to note that during the optimization, the design space for the connecting beams is fixed (390x390 µm2) for the GA; this enables objective comparison of different designs. It could be argued that for an orthogonal beam design, the actuation range can be improved by simply increasing the length of the connecting beam. However, in a fixed design space, the two adjacent orthogonal connecting beams will cross each other if the two connecting beams are prolonged beyond a certain level, which is physical obviously impossible. A serpentine orthogonal beam could be used to prevent this and prolong the beam length, however, this reduces the stiffness in the radial direction and thus increases R_m, leading to a low pull-in event. Therefore, constraining the design to a conventional orthogonal shape does not fully explore the design space and does not achieve an optimal design. More complex freeform geometrical shapes may result in a solution with superior performance. Thus, we propose to replace simple orthogonal structures with structures based on freeform geometries. Their shapes can be optimized with the GA to improve the actuation range at a low actuation voltage.

2.4 Figures of Merit (FOM)

In the following, we regard the sum of the displacements at the two gripper arm tips as the displacement of the microgripper, X_T. Ideally, a large X_T with a low actuation voltage is desired for an electrostatically actuated microgripper. Therefore, X_T for a fixed actuation voltage (40 V) was used as the FOM for the design process. The gap between the arm tips of the microgripper was designed as 100 µm, which obviously defines an upper limit for X_T. These values were chosen as most of the electrostatic microgrippers described in the literature require a voltage above 80 V to reach a X_T of 100 µm. Therefore, 40 V represents a typical mid-range actuation voltage, suitable for comparison.

Consequently, the GA is programmed in such a way that it maximizes X_T while maintaining R_m less than 1.3 µm.

2.5 Optimization Process

In the first step of the optimization process, the GA ran 40 individuals (i.e., designs with a specific parameter set), which were chosen randomly within the parameter ranges. For each individual, a FEM simulation was carried out for the fully parameterized mechanical model. The FEM simulation included a static displacement simulation for a fixed actuation voltage. For the simulation, the electro-mechanical multi-physics functionality in COMSOL was used, in which the electrostatic actuation force is calculated based on the number and geometry of the comb fingers, and the actuation voltage. The value of R_m > 1.3 µm or a converge failure of the simulation indicated a pull-in event. The

simulation result automatically was transferred to the GA in MATLAB, which recorded and sorted the results based on the FOM and performed several post processing steps. These included picking the ten best individuals (elite preservation), deriving a certain number of new random individuals (mutation) and cross-fertilizing good individuals to create new offspring. This last step involved taking different parameters from different good individuals and combining them to create a new individual (child). These three steps created the parameter value set for the 2nd generation. Then, the GA started the same optimization process for the 2nd generation, as for the 1st generation. For each simulation, a row of values was recorded and displayed in the command window of MATLAB.

In the 1st generation, the FOM varied considerably, indicating that the system still explored the design space. After the 1st generation, the GA already tended to find designs that have a large FOM. In the end, the GA consistently settled towards designs with a higher FOM and started to converge.

Fig. 3 shows a graphical illustration of the optimization process, which went through 8 generations. The GA changed the shape of connecting beams considerably. During the optimization, the GA attempted to make the connecting beams more compliant by bending them, to increase X_T. Also, the GA folded the connecting beams to increase their length, which further reduced the stiffness and improved X_T. However, due to the rotary comb actuator, the connecting beams would not only move along the tangential axis but also exhibited undesired movement along the radial axis, as illustrated in Fig. 2c. This increased the displacement of the microgripper in the R-axis in Fig. 2c. Thus, the GA attempted to reduce R_m by making the bends of two curved connecting beams face each other. In that way, the undesired movement of two curved connecting beams was in opposing directions and cancelled each other, reducing R_m. Finally, the undesired movement of the rotary comb actuator was reduced. (this will be discussed in detail in section V.C).

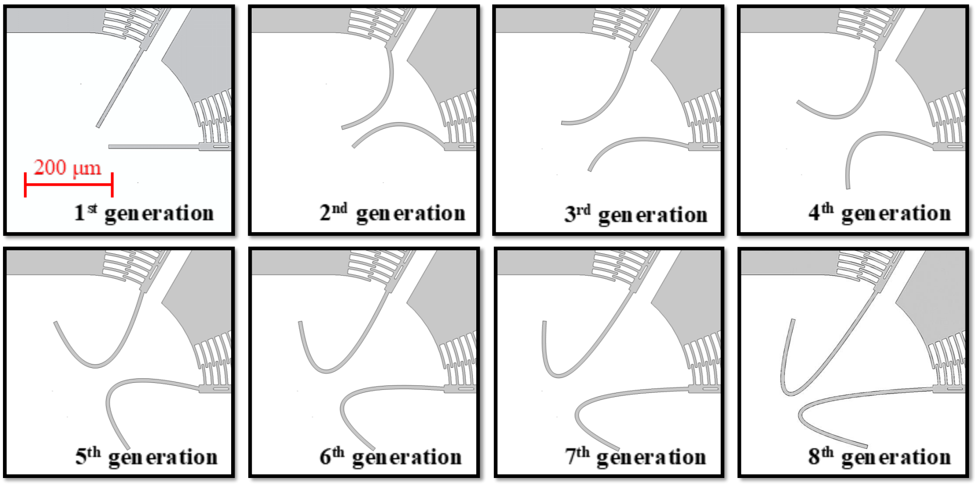

Fig. 3 Optimization process. The shape of connecting beams changes during the GA optimization.

2.6 Robustness analysis

The next step in the design process was a robustness analysis, which started by collecting 10 individuals with the highest FOM; these were taken as optimal design candidates.

For the robustness analysis, the designer had to specify a standard deviation of each design parameter representing the fabrication tolerances. 100 Gaussian distributed parameter sets were calculated of all parameters of an individual, using the mean value and designer supplied standard deviations. These effectively represent the fabrication tolerances. Therefore, for each individual, 100 simulations were run and the FOMs were recorded. A minimum threshold for the FOM was set by the designer. A yield value was calculated, representing the percentage of the simulations for each individual above the minimum FOM. The designer finally had to choose one as the final design by reviewing the yield and the FOM of the investigated individuals based on the application requirement.

3. Optimization Result

The GA optimization ran continuously for eight generations, with one generation size of 40 individuals, as shown in Fig. 3. As mentioned before, the minimum beam width for all designs was set to 7 μm during the optimization process. It is worth noting that the whole optimization process took 8 hours with a 3D mechanical model and 6 hours with a 2D mechanical model using a laptop with an i7 core of 2.5 GHz working frequency and 8 G RAM. The optimization was completed in 8 hours, with little manual intervention. The optimization process would take much less time if it ran on a work-station or in a parallel computation mode.

Two types of freeform designs were selected as the optimal designs, in the following referred to as CB7-D1 (Fig. 6b (1)) and CB7-D2 (Fig. 6b (2)); their parameter values are listed in Table 2. CB7-D1 had a larger X_T than CB7-D2. CB7-D2 had a larger R_m than CB7-D1. The difference between the two designs was mainly due to that CB7-D1 had a more compliant freeform beam than CB7-D2.

Table 2 definition, symbol, and upper and lower bounds of parameters

Parameter	CB7-D1	CB7-D2	SB7
GripperArmLength	1520 µm	1534 µm	1545 µm
GripperArmWidth	78 µm	88 µm	88 µm
ArmTipLength	180 µm	210 µm	190 µm
ArmTipWidth	79 µm	85 µm	90 µm
ArmTipAngle	35 °	35 °	35 °
FingerAngle	24 °	24 °	24 °
FingerAngleOffset	4 °	4 °	4 °
FingerLength	44 µm	44 µm	44 µm
FingerWidth	5 µm	5 µm	5 µm
FingerGap	3 µm	3 µm	3 µm
RotatryActuatorLength	940 µm	949 µm	960 µm
RotatryActuatorWidth	20 µm	18 µm	21 µm
ConnetingBeamLength	230 µm	221 µm	200 µm
ConnetingBeamWidth	7 µm	7 µm	7 µm
ConnetingBeamTopLength	210	198	238
ConnetingBeamTopWidthRatio	0.5	0.5	0.5
ConnetingBeamTopShiftX	5 µm	4 µm	0 µm
ConnetingBeamMiddleLengthRatio	0.6	0.7	0.5
ConnetingBeamMiddleWidthRatio	0.5	0.5	0.5
ConnetingBeamMiddleShiftX	25 µm	30 µm	0 µm
ConnetingBeamBottomWidthRatio	0.5	0.5	0.5
ConnetingBeamBottomShiftX	130 µm	112 µm	0 µm

To compare the freeform designs with a conventional orthogonal design, the same GA optimization algorithm was also run with constraints allowing only an orthogonal design. An identical design space (390x390 µm2) for chosen for the connecting beams to allow an objective comparison. The optimal orthogonal design was termed SB7 ((Fig. 6b (3)); Table 3 also lists its FOM. Compared with CB7-D1 and CB7-D2, SB7 had the lowest FOM. Here, 80% of the FOM value in each optimal design was taken as the minimum threshold of acceptable FOM values during the robustness analysis.

Table 3 The FOMs and simulated yield of the microgripper design CB7-D1, CB7-D2, SB7

Performance	FOM	Yield (%)
CB7-D1	59 µm	80
CB7-D2	49 µm	79
SB7	24 µm	87

According to the robustness analysis, CB7-D1, CB7-D2 and SB7 had a yield of 86% (minimum FOM of 47 µm), 84% (minimum FOM of 39 µm), 90% (minimum FOM of 19 µm), respectively.

As a freeform design has many degrees of freedom, it is necessary to disperse the parameter values during the optimization to achieve a global rather than a locally optimal solution. However, a too dispersed parameter space makes the optimization process computationally intensive. To study the convergence, the GA carried out 10 independent optimization processes by using different initial designs across the design space. As circumstantial evidence, the topologies of 10 optimal solutions resembled each other, indicating a global convergence of the optimization process to a large extent. The FOMs of the designs obtained in 10 different optimization runs ranged from 47 µm to 60 µm.

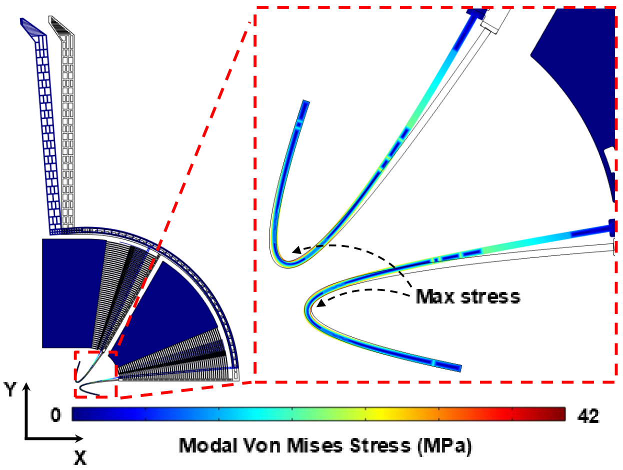

Fig. 4 Optimization result. Von Mises stress contour plot of the optimal freeform design CB7-D1 with an actuation voltage of 53 V and a X_T of 100 μm.

3.1 Static Analysis

According to a FEM simulation in COMOL, the freeform design CB7-D1 had a X_T of 100 μm for a DC actuation voltage of 53 V, as shown in Fig. 4. The freeform design CB7-D2 had a X_T of 100 μm for a DC actuation voltage of 57 V. The optimized orthogonal design SB7 had a X_T of 41 μm for a DC actuation voltage of 53 V and 48 μm for a DC actuation voltage of 57 V. It required a DC actuation voltage of 85 V for the orthogonal design SB7 to reach a X_T of 100 μm. Comparing the freeform design CB7-D1 with the orthogonal design SB7, X_T was increased by 144% for the same DC actuation voltage of 53 V, as shown in Table 4. Besides, the actuation force for the freeform design CB7-D1 to reach a X_T of 100 μm was only 39% of that of the orthogonal design SB7, as shown in Table 4. Therefore, the stiffness of the connecting beams in the freeform design CB7-D1 is lower than that in the orthogonal design SB7. The output force of the gripper when grasping a micro-object is an important parameter of the gripper performance, which is directly related to the stiffness of the connecting beams. The freeform design CB7-D1 is thus expected to be less harmful to fragile samples during manipulation compared with the orthogonal design SB7.

Table 4 The X_T of the microgripper design CB7-D1, CB7-D2, SB7 under different actuation voltages

DC actuation voltage (V)	53	57	85
CB7-D1 X_T (μm)	100	/	/
CB7-D2 X_T (μm)	83	100	/
SB7 X_T (μm)	41	48	100

3.2 Dynamic Analysis

Given the significant influence of vibration modes and stress on the microgripper, these parameters were analyzed next. The frequencies of the first three modes of the freeform design CB7-D1 were 823 Hz, 10583 Hz and 27932 Hz, respectively. The 2nd mode frequency is 11.86 times larger than the working mode (1st mode) frequency, which dramatically increased the stability during actuation. The frequencies of the first three modes of the freeform design CB7-D2 and the orthogonal design SB7 are listed in Table 5; the mode shapes of CB7-D1 were very similar.

Table 5 First three modes of the microgripper design CB7-D1, CB7-D2, SB7

Design	1st Mode (Hz)	2nd Mode (Hz)	3rd Mode (Hz)
CB7-D1	823	10583	27932
CB7-D2	906	11484	29365
SB7	1245	16975	48530

3.3 Stress Analysis

In our design, the connecting beams are used to support the movable structures and to bend during a gripping operation. That makes the connecting beams the most fragile part of the design and thus, they could break under a large electrostatic force input. Hence a stress analysis was performed to predict the stress distribution of the microgripper during actuation. According to a FEM simulation in COMSOL, when the freeform microgripper CB7-D1 reached 100 μm (its maximum X_T), the maximum Von Mises stress was 42 MPa (as shown in Fig. 4), which is much smaller than the yield strength of single-crystal silicon, i.e., 7 GPa [28]. This low stress value is another benefit of the freeform geometries and the GA optimization. Compared with orthogonal beams, stress can be more evenly distributed by the

curved shapes of freeform beams and stress concentration can be prevented. Also, the GA attempted to reduce the stress to increase the X_T, since a low stress concentration leads to a large X_T. As shown in Fig. 4, the stress was evenly distributed on the freeform. As will be discussed later, in the experiment, none of the microgrippers broke during actuation. Furthermore, the microgripper did not break even when we manually probed the arm tips of the microgripper to release them from the actuation combs after a pull-in event. As shown in Fig. 4, the maximum Von Mises stress of CB7-D1 was located at the turning point of the freeform beam. The maximum Von Mises stress of the microgripper design CB7-D2 and design SB7 were 44 MPa and 179 MPa, respectively, when they reached a X_T of 100 µm.

4. Fabrication

Fig. 5 shows the SOI-based process flow used in this work, which is similar to the one described in [26]. After etching a pattern of frame trenches on the handle layer of a wafer by deep reactive-ion etching (DRIE), another pattern of trenches and etch holes were etched on the front side in a 50-µm-thick device layer. The handle layer beneath the rotary comb actuators, gripper arms as well as arm tips were removed to increase yield and reliability by offsetting the two trench patterns by 40 µm. Finally, the devices were separated from each other by HF vapor phase etching without the usage of a dicing step.

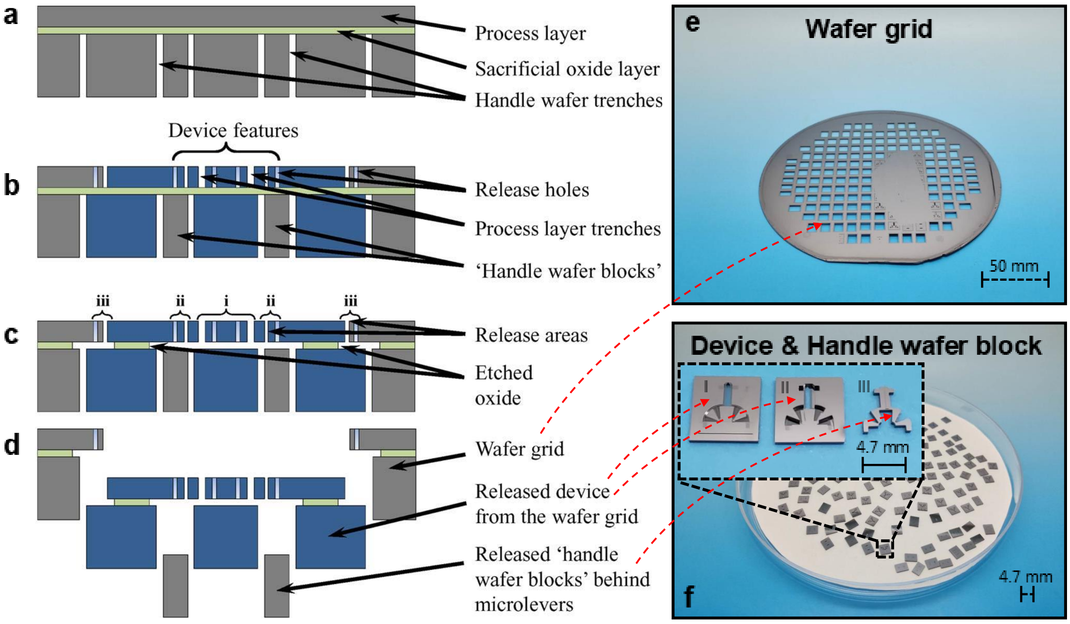

Fig. 5 Fabrication process. Fabrication flow of the MEMS accelerometers: **a** Backside etching using DRIE to define the backside trenches. **b** Front side DRIE to pattern the device features, release holes, and front side trenches. **c** Three release regions, namely, (i) device, (ii) handle wafer block release features, and (iii) dicing features, were etched consecutively by hydrofluoric acid in the vapour phase. **d** Device separation after release [26]. **e** image of the wafer grid of step **f** (the solid area resulted from a lithography fault). (f) image of the released devices. (I) The front image of the released device, (II) back image of the released device, and (III) released 'handle wafer blocks.

Fig. 6a shows the fabricated microgripper CB7-D1 with the curved shapes of the freeform beams. For designs CB7-D2 and SB7, the structure was identical to CB7-D1, except for the connecting beams. A comparison of the connecting beams of CB7-D1, CB7-D2 and SB7 is shown in Fig. 6b. We fabricated 172 chips on a 4-inch wafer, including freeform and orthogonal designs with a chip size of 3.7x3.7 mm2. 90% ~ 95% of all fabricated chips had complete structures and were fully functional after releasing, bonding, and packaging. This fabrication result indicated the yield rate of the freeform MEMS devices was as good as of the orthogonal MEMS designs as long as rules concerning minimum feature size (such as minimum etching trenches, minimum widths) were adhered.

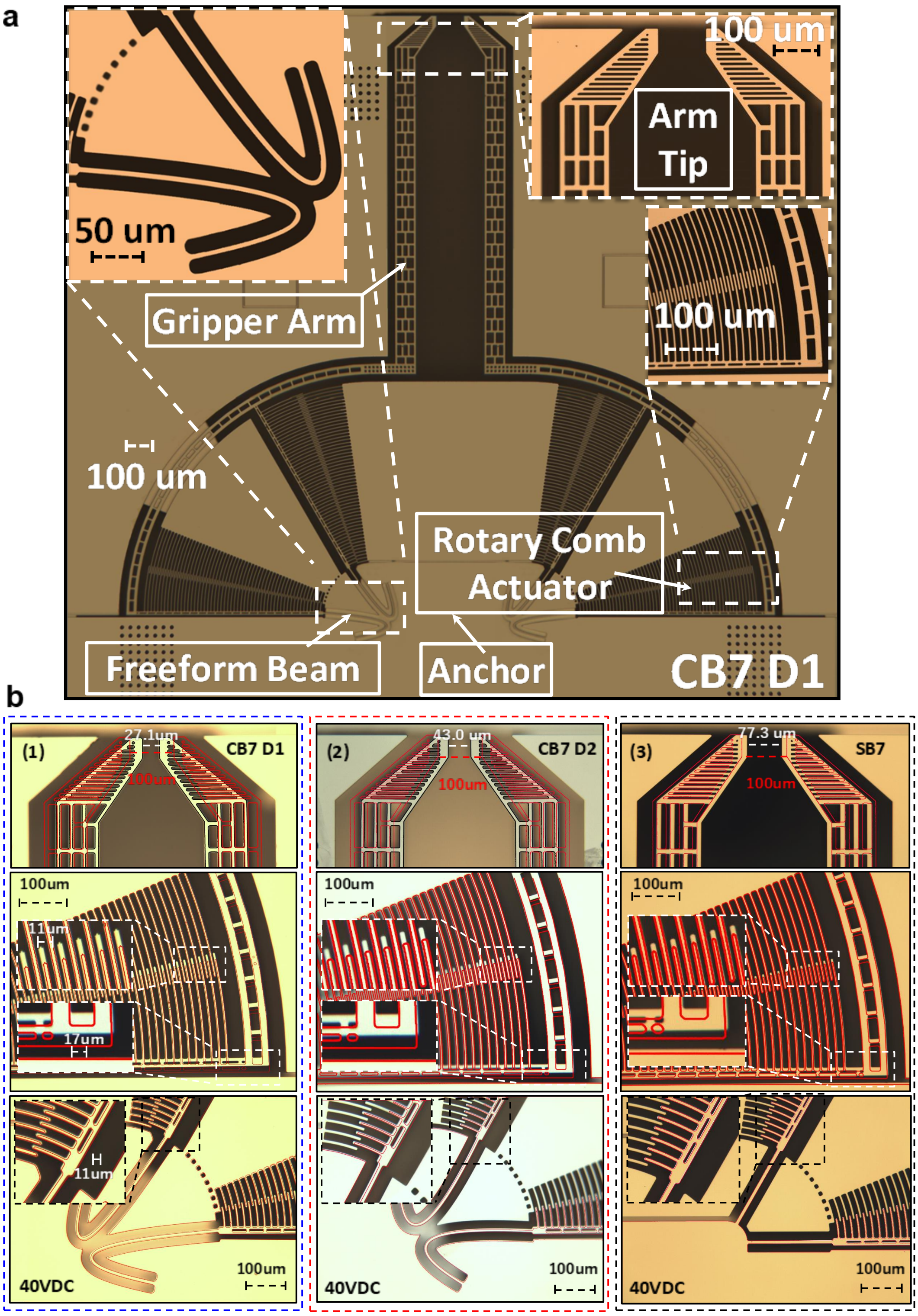

Fig. 6 Fabricated microgrippers and their displacement under 40 V actuation. a The metallographic microscope image of the freeform microgripper CB7-D1. **b** The images of the microgripper CB7-D1, CB7-D2 and SB7 under a certain actuation voltage. Image (1), (2), (3). The images of the microgripper CB7-D1, CB7-D2 and SB7 under an actuation voltage of 40 V. For comparison, the red contours indicate the position of the structure before actuation. The upper images show the arm tip area, the middle show the rotary comb actuator area and the bottom images show the connecting beam area. (1) CB7-D1, (2) CB7-D1 and (3) SB7 for an actuation voltage of 40 V. For comparison, the red contours indicate the position of the structure before actuation. The upper images show the arm tip area. The middle images show the rotary comb actuator area. The bottom images show the connecting beam area.

5. Experiment Results and Discussion

5.1 Experiment setup

As shown in Fig. 7a, the measurement setup included a voltage source, a multimeter, a microscope with a camera as well as an electronic circuit. The voltage source could supply a DC voltage ranging from 0 to 60 V. The multimeter was used to measure the exact voltage supplied to the microgripper. The microscope with a camera was used to measure the displacement and gripping action of the microgripper. The electronic circuit included some protecting resistors in case pull-in occurred and the current would become too high.

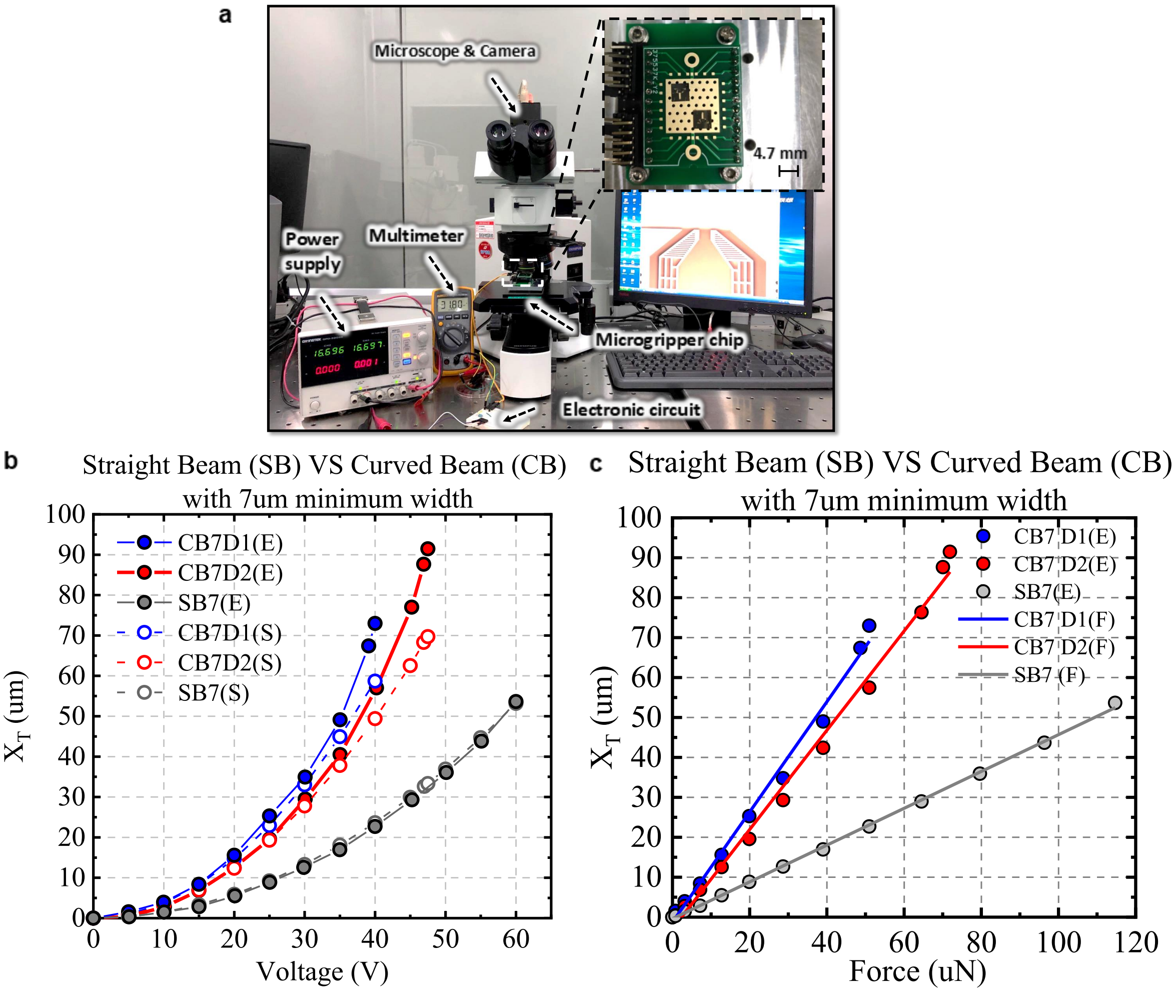

Fig. 7 Measurement setup and result. a Measurement setup. **b** Characterization of the simulated and measured X_T versus actuation voltages in two freeform designs (CB7-D1 (blue line), CB7-D2 (red line)) and one orthogonal design (SB (black line)). Simulated results (dashed lines): CB-7D1(S), CB7-D2(S), SB7(S). Measured results (solid lines): CB7-D1(E), CB7-D2(E), SB7(E). **c** Characterization of the simulated and measured X_T versus actuation force in two freeform designs (CB7-D1 (blue), CB7-D2 (red)) and one orthogonal design (SB (black)). Linear fitting lines (solid lines): CB-7D1(F), CB7-D2(F), SB7(F). Measured results (dots): CB7-D1(E), CB7-D2(E), SB7(E).

5.2 Gripping range test result

First, the gripping ranges were tested. Two types of freeform designs, i.e., CB7-D1 (blue line), CB7-D2 (red line)) and one orthogonal design, i.e., SB (black line) were tested. When different voltages were applied to the microgrippers, the images of the arm tips were acquired and processed to calculate the displacement. In Fig. 7b, the experimental results of three types of microgrippers are shown with a solid line, i.e., CB7-D1(E), CB7-D2(E), SB7(E). The experimental results indicated that the microgripper design CB7-D1 provided a gripping range of 73 µm with an actuation voltage of 40 V and design CB7-D2 gripping range of 91.5 µm with an actuation voltage of 47.5 V. Limited by the maximum voltage of the voltage source, microgripper design SB7 provided a gripping range of 48.0 µm with an actuation voltage of 60 V. Since the orthogonal design SB7 is only used to evaluate the improvement of the freeform designs CB7-D1 and CB7-D2 under the same actuation voltage, 60V was sufficient for testing design SB7.

In Fig. 7b，the simulated results of respective designs are also plotted (dashed lines), i.e., CB7-D1(S), CB7-D2(S) and SB7(S). The experimental results agree well with the simulation results. The small discrepancy is due to fabrication tolerances of the gripper parameters and the pull-in effect.

The displacements of the microgrippers were compared not only at the arm tips but also in the areas of the rotary comb actuators and connecting beams. A comparison of the three types of microgripper designs with an actuation voltage of 40 V is shown in Fig. 6b (1), (2), (3), in which the red contours indicate the position of the structure before

actuation. The X_T of design CB7-D1 was larger than that of design CB7-D2, which, in turn, was larger than that of design SB7 in all three areas. Since CB7-D1 could not be actuated higher than 40 V (which is close to the pull-in voltage) the comparison of designs CB7-D2 and SB7 was made with an actuation voltage of 47.5 V. The X_T of design CB7-D2 was much larger than that of the design SB7 in all three comparison areas. In summary, under the same actuation voltage, microgrippers with freeform geometries improved the X_T by 150-200% compared with orthogonal geometries in the same die area.

Fig. 7c shows the relationships between the actuation force and X_T for the three designs, i.e., CB7-D1, CB7-D2, SB7. The linear fittings were plotted using the least squares method. In terms of the connecting beam stiffness, CB7-D1 has a nonlinearity of 5.5% at the worst case for a 51 μN actuation force range; CB7-D2 has a nonlinearity of 5.6% at the worst case for a 72 μN actuation force range; SB7 has a nonlinearity of 2.2% at the worst case for a 115 μN actuation force range. CB7-D1, CB7-D2, SB7 have a nonlinearity of 5.5%, 5.2%, 1.2% at the worst case for a 51 μN actuation force range, respectively. As shown in Fig. 7c, among the three designs, SB7 has the lowest nonlinearity of the connection beams stiffness under the same actuation force range, as SB7 has the highest stiffness (the smallest X_T under the same actuation force). CB7-D2 and CB7-D1 have the 2nd and the 3rd lowest nonlinearity of the connection beams stiffness under the same actuation force. Thus, the higher the connecting beam stiffness is, the lower nonlinearity of the connecting beam stiffness under the same actuation force is.

According to the simulation, the total capacitance of the rotary comb actuators in CB7-D1 changes from 2.28 pF to 3.16 pF, after achieving a deflection X_T of 72.9 μm. The total capacitance of the rotary comb actuators in CB7-D2 changes from 2.28 pF to 3.28 pF, after achieving a deflection X_T of 91.5 μm. The total capacitance of the rotary comb actuators in S7-D2 changes from 2.28 pF to 3.00 pF, after achieving a deflection X_T of 54 μm. The effect of the fringing field does not play an important role and can be ignored during the actuation process [8,10].

5.3 Pull-in of rotary comb drives

It was found that for design CB7-D1, an actuation voltage higher than 40 V led to the pull-in of the rotary comb actuators, as shown in Fig. 8a. For an actuation voltage of 40 V, R_m of SB7 was not observable, whereas the R_m of CB7-D2 was about two times smaller than that of CB7-D1. Besides, pull-in occurred when the gripper of design CB7-D1 moved 74 μm under an actuation voltage of 41 V. Pull-in occurred in design CB7-D1 due to R_m becoming too large, resulting from the undesired movement of the curved beam along the R-axis. As shown in Fig. 8a.(4), the out-most comb fingers had the largest R_m value compared with other comb fingers and reached the pull-in point first, as the long lever of the rotary comb actuator acts like a motion amplifier.

The pull-in effect can easily be mitigated by increasing the stiffness of the connecting beams along the R-axis (e.g., by increasing the beam width). However, this will reduce the X_T under a given actuation voltage. After optimization, design CB7-D2 reached a larger X_T (91.5 μm) with a higher pull-in voltage (47.5 V).

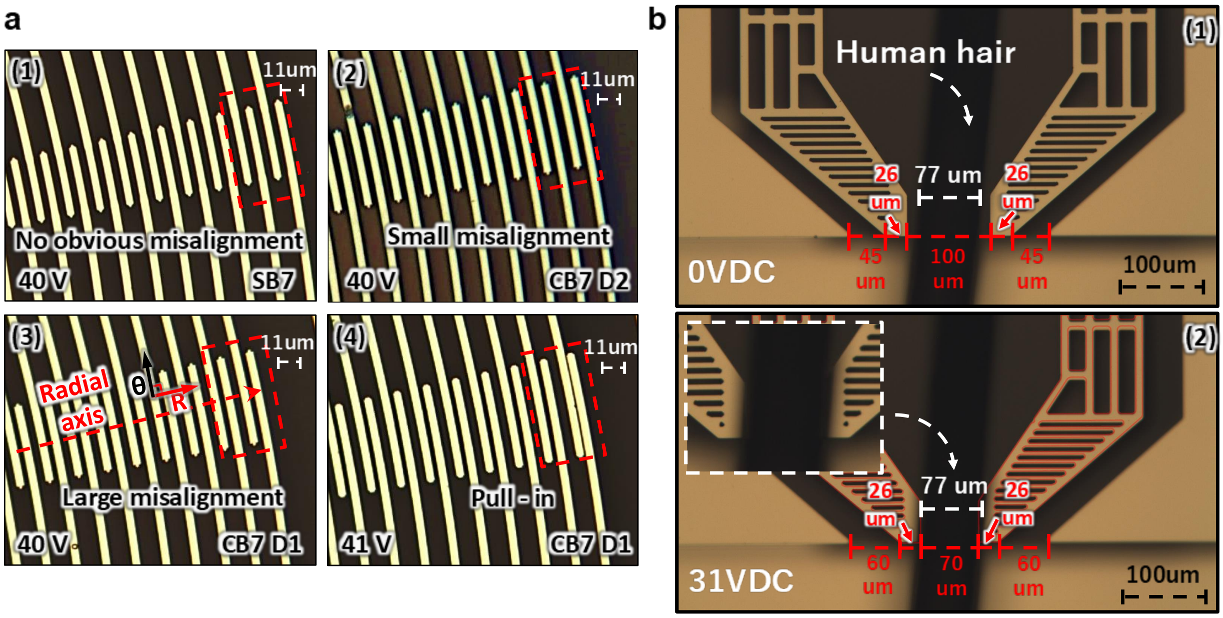

Fig. 8 **Results of 47.5 V actuation experiment, pull-in experiment as well as gripping experiment a** Images of designs CB7-D2 and SB7 under an actuation voltage of 47.5 V. Comparison of radial deflection R_m of the rotary comb actuators for different microgripper designs. (1) No apparent R_m in the microgripper design SB7 under an actuation voltage of 40 V. (2) Small R_m in the microgripper design CB7-D2 under an actuation voltage of 40 V. (3) Large R_m in the microgripper design CB7-D1 under an actuation voltage of 40 V. (4) Pull-in of the microgripper design CB7-D1 under an actuation voltage of 41 V. **b** Gripping a human hair with a diameter of 77 μm using microgripper design CB7-D2 (1) before gripping, (2) after gripping.

5.4 *Demonstration of Micro Object Gripping*

To demonstrate the performance of the fabricated microgripper, microgripper design CB7-D2 was used to grip a human hair with a diameter of 77 μm. The position of the microgripper relative to the hair before the gripping test is shown in Fig. 8b (1), in which the gap of the arm tips is 100 μm. It is worth noting that the micro stick-slip motion between the object and arm tip is mainly determined by the friction force. In [29], Zhang and Liu *et al.* found the micro stick-slip motion could be explained by Stribeck model, Dahl model, LuGre model. LuGre model has the best accuracy. The Coulomb friction model and the elastoplastic model do not work in a micro stick-slip motion system.

Then, the microgripper was driven with a voltage of 31 V and gripped the hair, as shown in Fig. 8b (2). The measured gap of the arm tips was 70 μm, smaller than the diameter of the hair, indicating the successful gripping of the hair. Also, according to Fig. 7b, CB7-D2 was expected to have a X_T of 30 μm for an actuation voltage of 31 V, matching the experimental result shown in Fig. 8b (2).

6. Discussion

For the same actuation voltage, microgrippers with freeform geometries (CB7-D1, CB7-D2) improved X_T by 150-200% compared with orthogonal geometries (SB7) for the same die area. Therefore, the use of freeform geometries has two practical advantages i) a lower actuation voltage to reach the same X_T, ii) less harm to fragile objects during gripping and releasing.

However, electrostatic rotary microgrippers exhibit an undesired radial displacement R_m during actuation. This leads to a reduction of the gap of the comb drive electrode, potentially causing pull-in, which limits the maximal X_T. With the proposed optimization method, R_m of the rotary comb actuators is included in the FOM. The GA based optimization concurrently maximizes X_T and minimizes R_m for a given voltage.

Comparing the two freeform designs, CB7-D1 has a larger X_T but a larger R_m compared with CB7-D2 for the same actuation voltage. Thus, a designer can select freeform designs according to different requirements for the gripping range. For example, for objects with a diameter between 100 μm and 30 μm, design CB7-D1 is superior to CB7-D2, as CB7-D1 can satisfy the gripping range with a lower actuation voltage. Also, for objects with a diameter between 100 μm and 10 μm, CB7-D2 is better than CB7-D1, as CB7-D1 can offer a larger gripping range while CB7-D1 pulls in after 74 μm.

Table 6 compares the gripping range of our microgrippers with those of other electrostatically actuated microgrippers in the literature. To compare the actuation ability of different designs fairly, the maximum X_T is divided by the square of the related actuation voltage and the calculated result is taken as the actuation ability. Crescenzi *et al.*'s design [9] has the highest actuation ability and lowest actuation force but a limited gripping range. Compared with Crescenzi *et al.*'s design, CB7-D1 actuation ability is 6 times lower, while the gripping range is 3.6 times larger, whereas the design CB7-D2 actuation ability is 5 times lower while the gripping range is 4.6 times larger.

Hao *et al.*'s design [8] has the second-highest actuation ability and largest gripping range. The high actuation ability in Hao *et al.*'s design is due to its narrow beam width (3.6 μm). If the beam width of CB7-D2 was reduced from 7 μm into 3.6 μm, a simulation indicated that CB7-D2 would only need 27 V to have a X_T of 100 μm, smaller than the 31 V of Hao *et al.*'s design. Moreover, the actuation voltage can be further reduced through the increase in the number of rotary comb actuators, since Hao *et al.*'s design has 6 groups of rotary comb actuators while our designs only have 4 groups of rotary comb actuators.

Also, the CB7-D1 and the CB7-D2 design were developed based on Chang *et al.*'s design [10]. Compared with Chang *et al.*'s design [10], CB7-D1 actuation ability is 4.8 times larger, while the gripping range is 1.4 times lower, whereas the design CB7-D2 actuation ability is 4.3 times larger while the gripping range is 1.1 times lower.

Table 6 Comparison of different grippers' operating displacement

Design	Actuation force (μN)	Actuation Voltage (V)	Actuation range (μm)	Actuation ability (nm/V^2)	Die Area (μm^2)	Gripper arm length (mm)
Volland [30]	231	80	20	3.13	1250*3300	1
Beyeler [22]	986	150	100	4.44	7700*5600	3.3
Chen [23]	1646	80	25	3.91	5745*3217	1
Bazaz [25]	1181	50	17	6.80	4891*6402	2.5
Chang [10]	297	100	94	9.40	3100*3700	1.7
Piriyanont [31]	104	80	90	14.06	8500*5600	1.6
Xu [32]	273	72	63	12.15	2800*3812	NA
Hao [8]	*37*	*31.5*	*100*	100.78	*4500*4000*	1.7
Crescenzi [9]	*3*	*11*	*20*	165.29	*2710*4417*	1.4
**(CB7-D1)**	**51***	**40**	**72.9**	45.54	**3700*3700**	**1.7**
**(CB7-D2)**	**72***	**47.5**	**91.5**	40.55	**3700*3700**	**1.7**
**(SB7)**	**115**	**60**	**54**	15.00	**3700*3700**	**1.7**

* from COMSOL simulation.

7. Conclusions

A novel microgripper with freeform geometries designed using a GA approach is presented. The GA based semi-automated design methodology with freeform geometries is introduced in detail. It is capable of designing near-optimal MEMS devices that are robust to fabrication tolerances. Two types of microgrippers with freeform geometries and one microgripper with orthogonal geometries were optimized by this method. FEA simulations were used to analyze the static and dynamic performance as well as the stress distribution of the designed microgrippers. The experiment showed that the microgripper with freeform geometries had a large X_T (91.5 μm) for a low actuation voltage (47.5 V), which agreed well with the theory. This made it possible to manipulate a wide range of objects (size ranging from 10 μm to 100 μm). The concept was successfully demonstrated by grasping a human hair with a diameter of 77 μm. A detailed analysis of the pull-in effect due to the R_m of the actuator electrodes was conducted. Possible methods to mitigate this effect were also discussed.

For the same actuation voltage, microgrippers with freeform geometries improved X_T by 150-200% compared with orthogonal geometries in the same die area. Thus, freeform geometries have two advantages i) a lower actuation power to reach the same X_T, ii) less harm to fragile objects during gripping and releasing.

From Table 6, we briefly compare our freeform geometries design with the two best electrostatic microgrippers described in the literature [8,9] in terms of actuation range and X_T per voltage2 (actuation ability). Both freeform geometries developed in this work have a larger gripping range compared to Crescenzi *et al.* [9]. If the same number of actuation comb fingers is considered our designs have a better actuation ability compared to Hao *et al.* [8].

The improved performance of the microgripper is mainly due to the use of GA for freeform geometric design. It is worth pointing out that the proposed design methodology enabling freeform geometries can be extended to a wide range of other MEMS devices. Future work will include equipping the microgripper with both force sensing and a feedback system. This will allow the gripping process to be performed with a higher precision more controllable force, creating the ability for fast, automated operation.

8. Acknowledge

This research was funded by the Science Challenge Project, grant number TZ2016006-0502-02, Natural Science Foundation of Hubei Province under Grant 2019CFB108.

9. Conflicts of Interest

The authors declare no conflicts of interest.

10. Author Contributions

C.W. designed, built, and tested the MEMS microgripper. W.F. and Y.W. contributed to the design of the MEMS mechanism. X.S., S.S., M.G. contributed to the MEMS fabrication. H.L. contributed to the measurement setup. C.W., W.F., Y.W. took the measurements. H.Z., C.W., Y.W, A.Q. performed computational analysis of the data. C.W., H.L., J.B., M.K. led the writing of the manuscript, and all authors provided comments. H.L., J.B., M.K. had the initial concept of the microgripper. H.L., J.B. oversaw the design, fabrication, and testing of the microgripper.

11. References

[1] Thornell, G., Bexell, M., Schweitz, J.-Å. & Johansson, S. Design and fabrication of a gripping tool for micromanipulation. *Sensors and Actuators A: Physical* **53**, 428-433 (1996).

[2] Ansel, Y., Schmitz, F., Kunz, S., Gruber, H. & Popovic, G. Development of tools for handling and assembling microcomponents. *Journal of Micromechanics and Microengineering* **12**, 430 (2002).

[3] Chu, L. L. & Gianchandani, Y. B. A micromachined 2D positioner with electrothermal actuation and sub-nanometer capacitive sensing. *Journal of Micromechanics and Microengineering* **13**, 279 (2003).

[4] Somà, A. *et al.* Design and experimental testing of an electro-thermal microgripper for cell manipulation. *Microsystem Technologies* **24**, 1053-1060 (2018).

[5] Xu, Q. Precision position/force interaction control of a piezoelectric multimorph microgripper for microassembly. *IEEE Transactions on Automation Science and Engineering* **10**, 503-514 (2013).

[6] Kim, D.-H., Lee, M. G., Kim, B. & Sun, Y. A superelastic alloy microgripper with embedded electromagnetic actuators and piezoelectric force sensors: a numerical and experimental study. *Smart materials and structures* **14**, 1265 (2005).

[7] AbuZaiter, A., Nafea, M. & Ali, M. S. M. Development of a shape-memory-alloy micromanipulator based on integrated bimorph microactuators. *Mechatronics* **38**, 16-28 (2016).

[8] Hao, Y., Yuan, W., Zhang, H., Kang, H. & Chang, H. A rotary microgripper with locking function via a ratchet mechanism. *Journal of Micromechanics and Microengineering* **26**, 015008 (2015).

[9] Crescenzi, R., Balucani, M. & Belfiore, N. P. Operational characterization of CSFH MEMS technology based hinges. *Journal of Micromechanics and Microengineering* **28**, 055012 (2018).

[10] Chang, H. *et al.* A rotary comb-actuated microgripper with a large displacement range. *Microsystem technologies* **20**, 119-126 (2014).

[11] Nielson, G. N. & Barbastathis, G. Dynamic pull-in of parallel-plate and torsional electrostatic MEMS actuators. *Journal of microelectromechanical systems* **15**, 811-821 (2006).

[12] Wang, C. *et al.* Micromachined accelerometers with sub-μg/√ Hz noise floor: A review. *Sensors* **20**, 4054 (2020).

[13] Howell, L. L. in *21st century kinematics* 189-216 (Springer, 2013).

[14] Middlemiss, R. *et al.* Measurement of the Earth tides with a MEMS gravimeter. *Nature* **531**, 614-617 (2016).

[15] Boom, B. A. *et al.* in *2017 IEEE 30th International Conference on Micro Electro Mechanical Systems (MEMS).* 33-36 (IEEE).

[16] Khan, S. & Ananthasuresh, G. Improving the sensitivity and bandwidth of in-plane capacitive microaccelerometers using compliant mechanical amplifiers. *Journal of microelectromechanical systems* **23**, 871-887 (2014).

[17] Krishnan, G. & Ananthasuresh, G. Evaluation and design of displacement-amplifying compliant mechanisms for sensor applications. (2008).

[18] Pedersen, C. B. & Seshia, A. A. On the optimization of compliant force amplifier mechanisms for surface micromachined resonant accelerometers. *Journal of Micromechanics and Microengineering* **14**, 1281 (2004).

[19] Cao, L., Dolovich, A. T., Schwab, A. L., Herder, J. L. & Zhang, W. Toward a unified design approach for both compliant mechanisms and rigid-body mechanisms: Module optimization. *Journal of Mechanical Design* **137**, 122301 (2015).

[20] Wang, C. *et al.* Design of freeform geometries in a MEMS accelerometer with a mechanical motion preamplifier based on a genetic algorithm. *Microsystems & Nanoengineering* **6**, 1-15 (2020).

[21] *COMSOL*, <https://www.comsol.com/ (accessed 23 May 2020)> (

[22] Beyeler, F. *et al.* Monolithically fabricated microgripper with integrated force sensor for manipulating microobjects and biological cells aligned in an ultrasonic field. *Journal of microelectromechanical systems* **16**, 7-15 (2007).

[23] Chen, T., Sun, L., Chen, L., Rong, W. & Li, X. A hybrid-type electrostatically driven microgripper with an integrated vacuum tool. *Sensors and Actuators A: Physical* **158**, 320-327 (2010).

[24] Piriyanont, B., Fowler, A. G. & Moheimani, S. R. Force-controlled MEMS rotary microgripper. *Journal of Microelectromechanical Systems* **24**, 1164-1172 (2015).

[25] Bazaz, S. A., Khan, F. & Shakoor, R. I. Design, simulation and testing of electrostatic SOI MUMPs based microgripper integrated with capacitive contact sensor. *Sensors and Actuators A: Physical* **167**, 44-53 (2011).

[26] Sari, I., Zeimpekis, I. & Kraft, M. A dicing free SOI process for MEMS devices. *Microelectronic Engineering* **95**, 121-129 (2012).

[27] Yeh, J. A., Chen, C.-N. & Lui, Y.-S. Large rotation actuated by in-plane rotary comb-drives with serpentine spring suspension. *Journal of Micromechanics and Microengineering* **15**, 201 (2004).

[28] Senturia, S. D. *Microsystem design*. (Springer Science & Business Media, 2007).

[29] Liu, Y., Li, J., Zhang, Z., Hu, X. & Zhang, W. Experimental comparison of five friction models on the same test-bed of the micro stick-slip motion system. *Mechanical Sciences* **6**, 15-28 (2015).

[30] Volland, B., Heerlein, H. & Rangelow, I. Electrostatically driven microgripper. *Microelectronic engineering* **61**, 1015-1023 (2002).

[31] Piriyanont, B. & Moheimani, S. R. MEMS rotary microgripper with integrated electrothermal force sensor. *Journal of Microelectromechanical Systems* **23**, 1249-1251 (2014).

[32] Xu, Q. Design of a large-range compliant rotary micropositioning stage with angle and torque sensing. *IEEE Sensors Journal* **15**, 2419-2430 (2014).
